# Supplementary material for: Structural Lipids Enable the Formation of Functional Oligomers of the Eukaryotic Purine Symporter UapA
Source: Cell Chem Biol. 2018 Jul 19;25(7):840–848.e4. doi: 10.1016/j.chembiol.2018.03.011 (PMC6058078; doi:10.1016/j.chembiol.2018.03.011)
Supplement: Document S1. Figures S1–S5 and Table S1 [file mmc1.pdf]

Cell Chemical Biology, Volume *25*

Supplemental Information

Structural Lipids Enable the Formation of Functional Oligomers of the Eukaryotic Purine Symporter UapA

Euan Pyle, Antreas C. Kalli, Sotiris Amillis, Zoe Hall, Andy M. Lau, Aylin C. Hanyaloglu, George Diallinas, Bernadette Byrne, and Argyris Politis

Supplementary Figure Titles and Legends

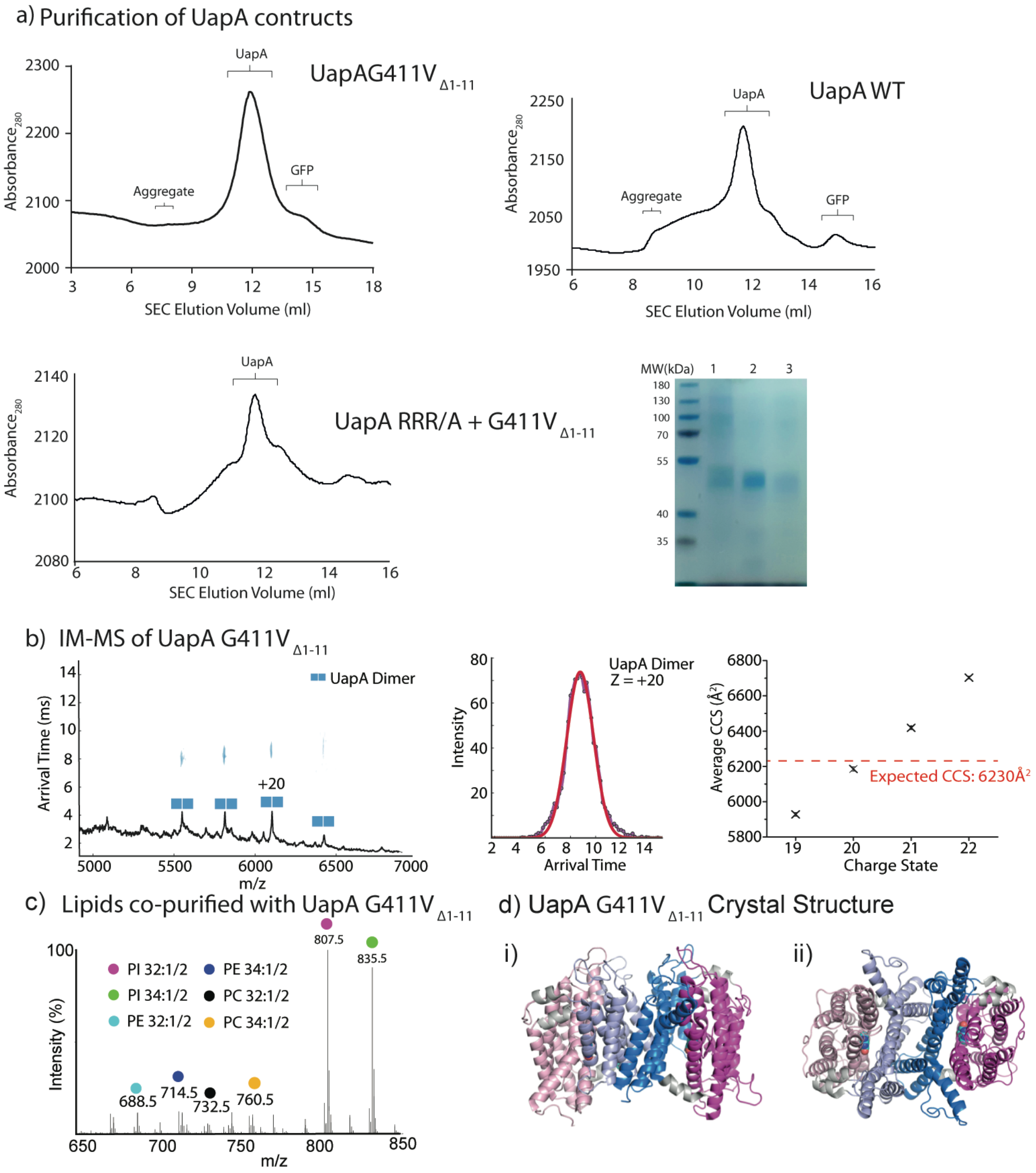

Figure S1, related to Figure 1 a) SEC elution profile of UapAG411V$_{\Delta 1\text{-}11}$, UapA RRR/A + G411V$_{\Delta 1\text{-}11}$, and WT UapA (λ=280 nm) with an SDS-PAGE gel showing the purity of UapA after SEC (lane 1: WT UapA, lane 2: UapAG411V$_{\Delta 1\text{-}11}$, lane 3: UapA RRR/A + G411V$_{\Delta 1\text{-}11}$) (lanes without protein loaded into their respective wells have been digitally cropped) (b) Ion-Mobility MS analysis of the UapAG411V$_{\Delta 1\text{-}11}$ dimer. IM-MS measurements were taken at a trap CE of 20 V and a transfer CE of 200 V. The resulting spectrum (left) is displayed with the arrival time distribution of z = +20 charge state shown (centre). The average CCS of each charge state (n= 3) ($\pm$ s.d.) was plotted against the expected CCS of UapAG411V$_{\Delta 1\text{-}11}$ calculated by analysing the UapAG411V$_{\Delta 1\text{-}11}$ crystal structure (PDB: 5I6C) (Alguel et al. 2016) using MOBCAL software (Shvartsburg and Jarrold 1996). (c) Negative ion mass spectrum of

UapAG411V$_{\Delta 1\text{-}11}$ in the low m/z range identifying the lipids co-purified with UapAG411V$_{\Delta 1\text{-}11}$. Due to overlapping peaks of mono-unsaturated and poly-unsaturated fatty acid chains, both species are identified by 1/2 notation. d) Structure of UapA (PDB: 5I6C) highlighting the domain organisation of the protein. The UapA dimer is shown (i) looking through the membrane and (ii) from the extracellular side of the membrane. The core domains of the individual monomers are coloured in light pink and magenta and the gate domains are coloured in light blue and dark blue. The amphipathic helices connecting the core and gate domains are coloured in gray and xanthine is shown in a space filling representation with cyan coloured carbon atoms.

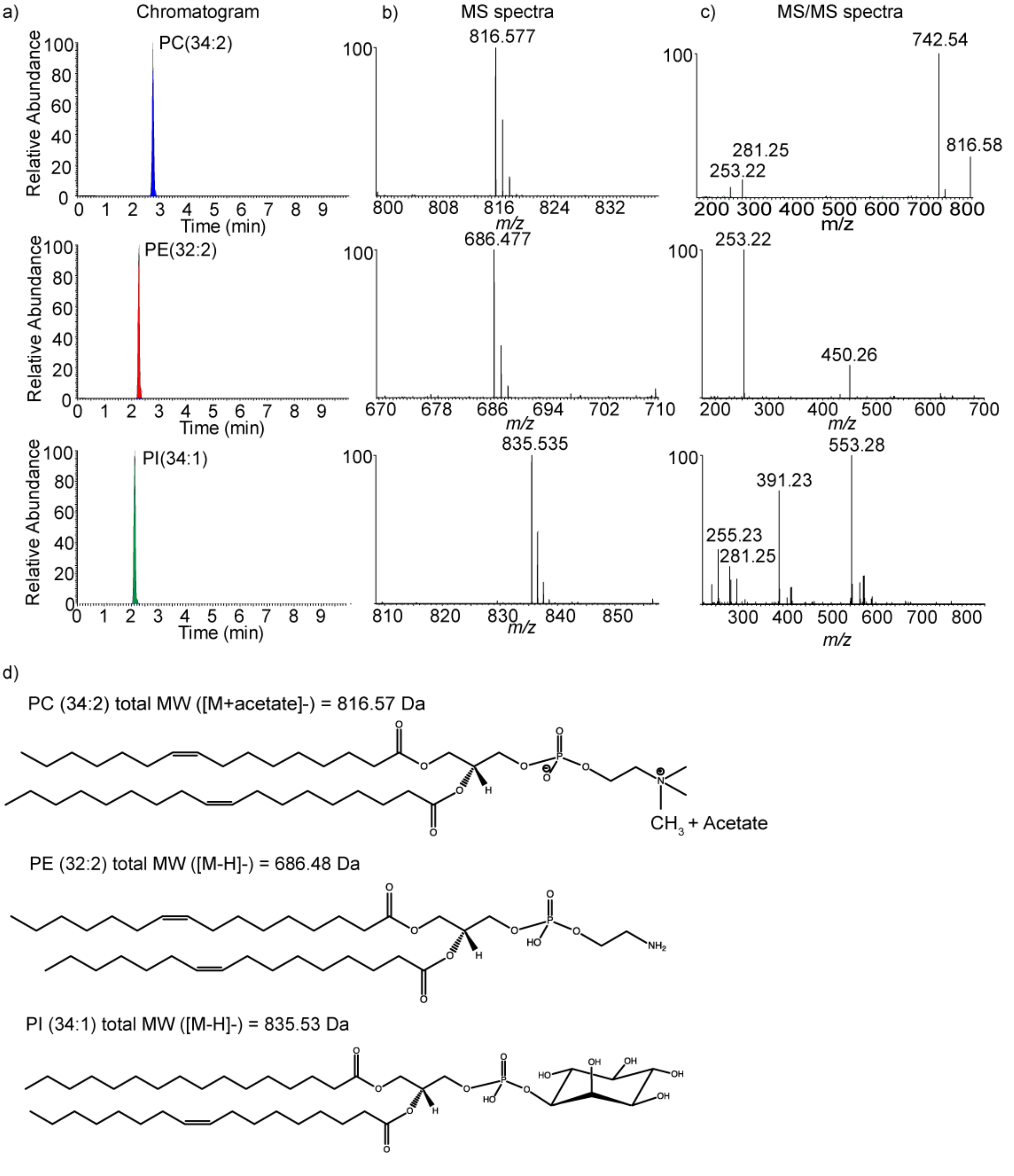

Figure S2, related to Figure 3 Results from LC-MS(/MS) analysis of UapAG411V$_{\Delta 1\text{-}11}$. LC traces (a) show the retention times of each major lipid species identified. The corresponding MS spectra (b) from the precursor ions of each lipid species are also displayed. LC-MS/MS spectra (c) show the fragmentation patterns of the lipid precursor ions. d) Molecular structure for each major lipid species identified by LC-MS(/MS). Also see **Table S1**.

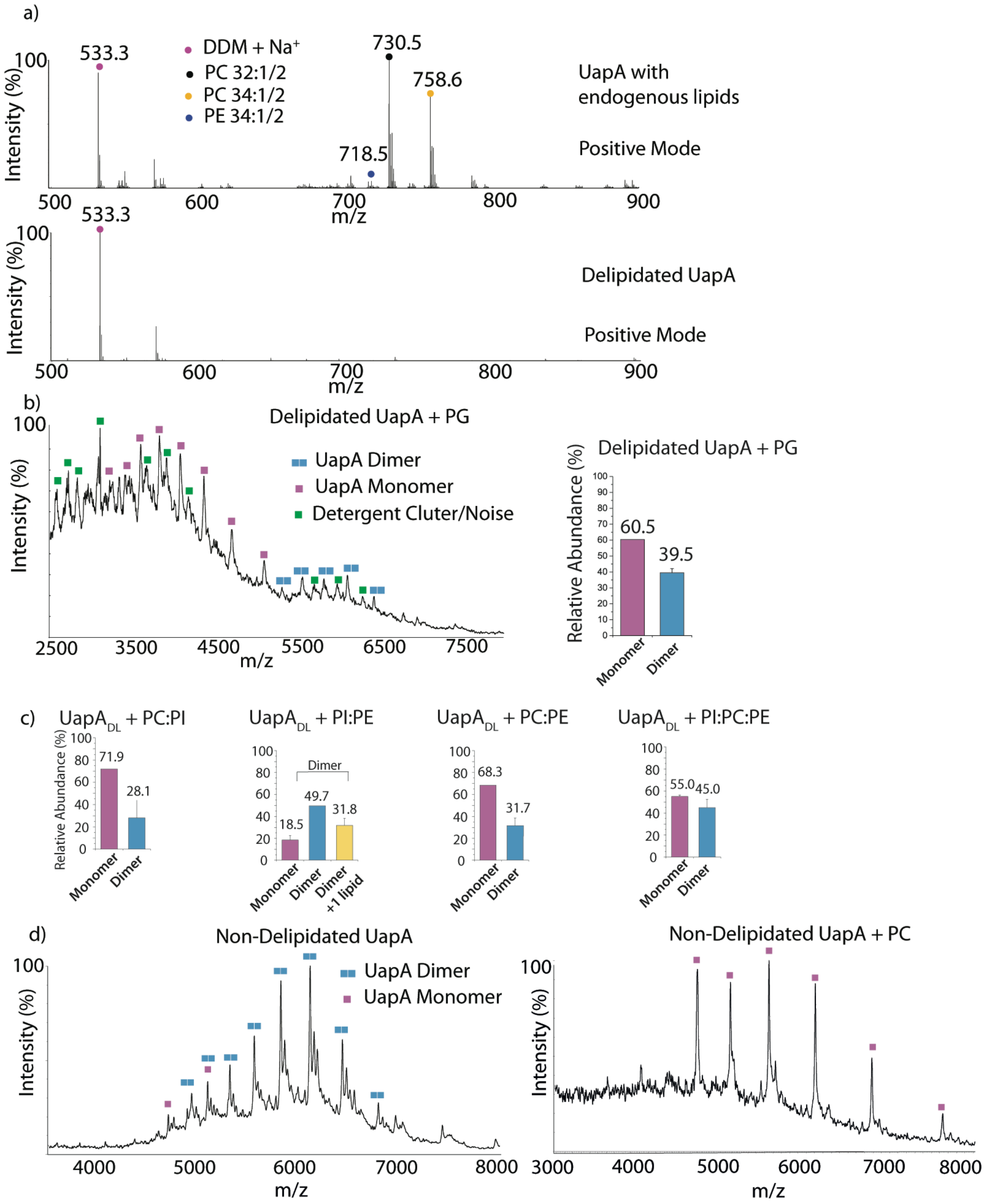

Figure S3, related to Figure 2 a) Spectra showing the presence of lipids in the low m/z range of a non-delipidated UapAG411V$_{\Delta 1\text{-}11}$ sample (top panel) in native MS and the loss of lipids in the same m/z range for a UapAG411V$_{\Delta 1\text{-}11}$ sample after delipidation (bottom panel). These spectra were recorded at a trap CE of 200 V and a transfer CE of 240 V. (b) Spectrum showing the effect of adding PG to delipidated UapAG411V$_{\Delta 1\text{-}11}$ at a ratio of 100:1 PG:UapA. The

relative abundance of each oligomeric state (right panel) of UapAG411V$_{\Delta 1\text{-}11}$ has been calculated using UniDec software. Data shown is the average ± s.d. Data is representative of three independent experiments carried out under identical conditions. This spectrum was recorded at a trap CE of 200 V and a transfer CE of 240 V. (c) Bar charts displaying the relative abundances of UapA species after the addition of equimolar mixtures of lipids to delipidated UapA. The relative abundances of each oligomeric species were quantified using UniDec software. Data shown is the average ± s.d. Data is representative of three independent experiments carried out under identical conditions. (d) Spectrum showing the effect of adding PC to non-delipidated UapAG411V$_{\Delta 1\text{-}11}$ at a ratio of 100:1 PC:UapA. These spectra were recorded at a trap CE of 200 V and a transfer CE of 240 V.

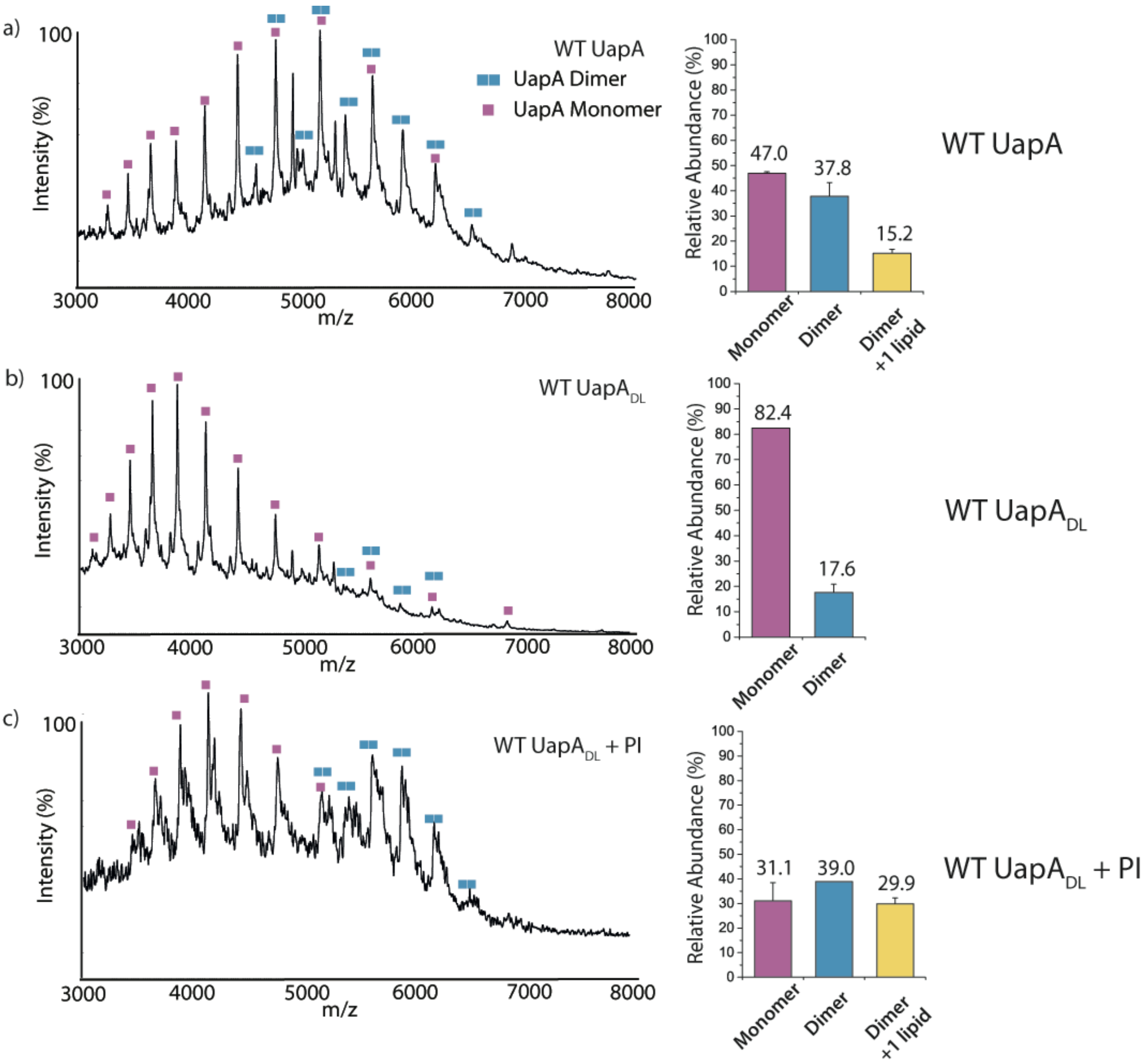

Figure S4, related to Figure 2 (a) Analysis of WT UapA, (b) delipidated WT UapA and (c) delipidated WT UapA following addition of PI. The left-hand panels show the spectra for the individual samples and the right hand panels show the relative abundance of the different oligomeric states obtained in each case. The spectra were recorded at a trap CE of 200 V and a transfer CE of 240 V. Quantification of the relative abundance of each oligomeric species was carried out using UniDec software. Data shown is the average ± s.d. Data is representative of three independent experiments carried out under identical conditions.

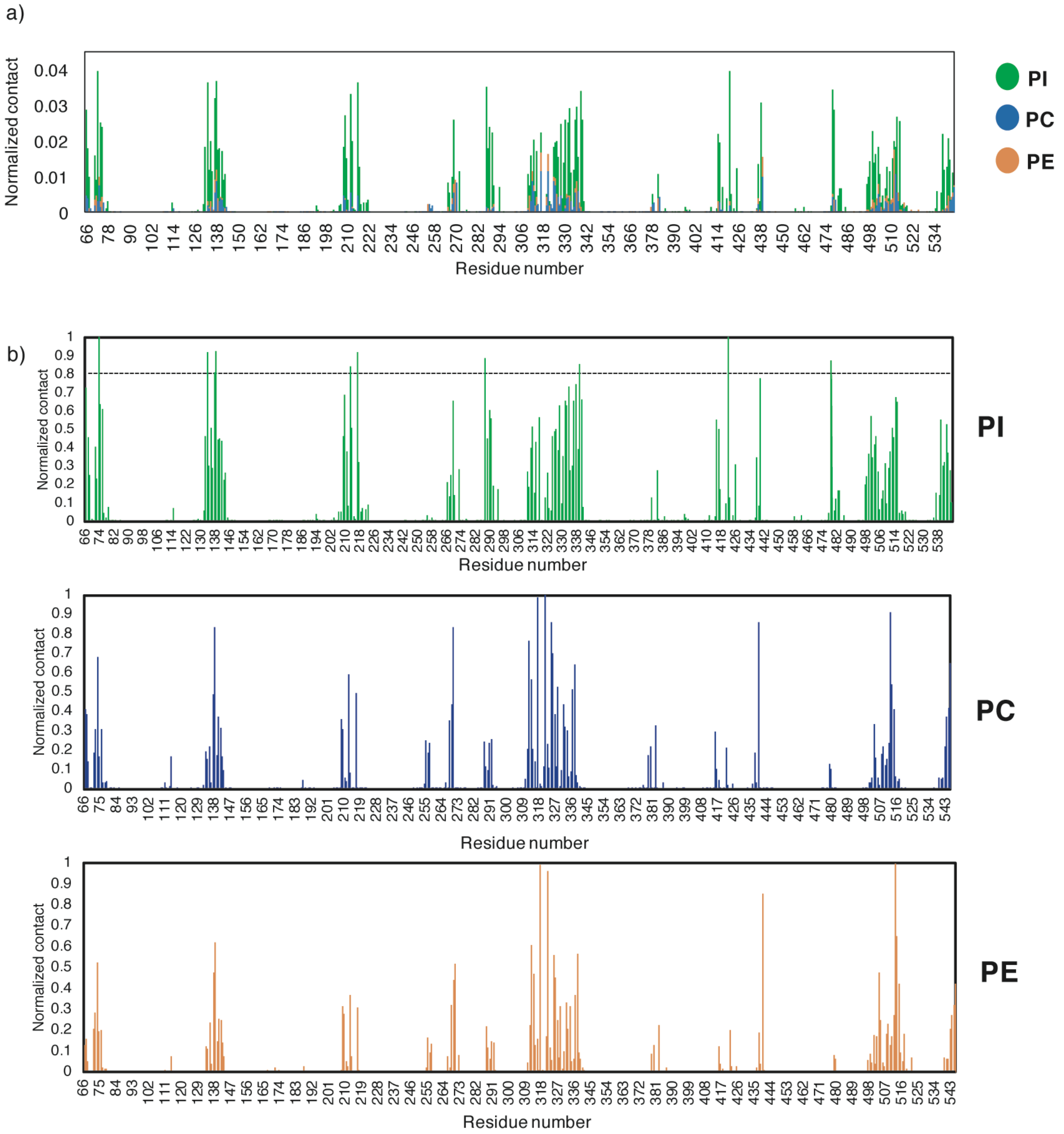

Figure S5, related to Figure 3: (a) Normalised average number of contacts (using a cut-off distance of 5.5 Å) between each UapA residue and the head groups of PI, PE, and PC lipids in the lipid bilayer in our MD simulations with a 40% POPC-25% POPE-35% PI bilayer (no restraints in the UapA interface). Each peak represents the probability of a residue to interact with each lipid type over the 5 µs of the simulations. For the normalization, the number of contacts of a residue with a lipid type was divided by the number of lipids, the number of frames and the number of lipids in the simulation. (b) Normalised average number of contacts between each UapA residue and the head groups of PI, PE, and PC lipids in the lipid bilayer of

our MD simulations. For the normalization, the number of contacts of a residue with the lipids was divided by the largest number of contacts, the number of frames and the number of lipids in the simulation.

Lipid	Acyl chains	Species	Lipid Mass (Neutral)	**Mean Lipid Abundance (mol) (%)**
PC(32:2)	16:1/16:1	$[M+acetate]^-$	729.5309	**13.6 ± 2.8**
PC(32:1)	16:0/16:1	$[M+acetate]^-$	731.5465	**6.6 ± 3.1**
PC(34:1)	16:0/18:1	$[M+acetate]^-$	759.5778	**4.3 ± 1.7**
PC(34:2)	16:1/18:1	$[M+acetate]^-$	757.5622	**10.9 ± 1.8**
PE(32:1)	16:0/16:1	$[M-H]^-$	689.4996	**6.0 ± 2.7**
PE(32:2)	16:1/16:1	$[M-H]^-$	687.4839	**4.1 ± 0.4**
PE(34:1)	16:0/18:1	$[M-H]^-$	717.7309	**6.6 ± 1.8**
PE(34:2)	16:1/18:1	$[M-H]^-$	715.5152	**5.5 ± 2.0**
PE(36:2)	18:1/18:1	$[M-H]^-$	743.5465	**3.0 ± 1.3**
PI(32:1)	16:0/16:1	$[M-H]^-$	808.5102	**10.5 ± 1.6**
PI(32:2)	16:1/16:1	$[M-H]^-$	806.4945	**3.5 ± 1.4**
PI(34:1)	16:0/18:1	$[M-H]^-$	836.5415	**13.9 ± 5.2**
PI(34:1)	16:1/18:1	$[M-H]^-$	834.5258	**4.6 ± 2.3**
PI(36:1)	18:0/18:1	$[M-H]^-$	864.5728	**1.9 ± 1.5**
CL(68:4)	16:1/16:1/18:1/18:1	$[M-H]^-$	1400.9722	**1.6 ± 0.9**
CL(68:4)	16:1/16:1/18:1/18:1	$[M-2H]^{2-}$	1400.9722	**2.0 ± 1.1**
CL(68:4)	16:1/16:1/18:1/18:1	$[M-2H+Na]^-$	1400.9722	**0.7 ± 0.7**
CL(70:4)	16:1/18:1/18:1/18:1	$[M-H]^-$	1429.0035	**0.4 ± 0.4**
CL(72:4)	18:1/18:1/18:1/18:1	$[M-H]^-$	1457.0348	**0.4 ± 0.4**

Green	Confirmed by MS/MS
Orange	Some diagnostic ions detected by MS/MS
Red	No diagnostic ions detected by MS/MS

Table S1, related to Figure 1: Table containing all lipids identified in a UapAG411V$_{\Delta 1\text{-}11}$ sample by LC-MS. Lipids confirmed by LC-MS/MS fragmentation are highlighted in green, lipids with incomplete fragmentation patterns in LC-MS/MS are highlighted in orange, and lipids with no matching fragmentation patterns in LC-MS/MS are highlighted in red. Lipid abundances displayed are the average $\pm$ s.d (n=3)
