# Supplementary material for: Structural Lipids Enable the Formation of Functional Oligomers of the Eukaryotic Purine Symporter UapA
Source: Cell Chem Biol. 2018 Jul 19;25(7):840–848.e4. doi: 10.1016/j.chembiol.2018.03.011 (PMC6058078; doi:10.1016/j.chembiol.2018.03.011)
Supplement: Document S2. Article plus Supplemental Information [file mmc2.pdf]

Article

Cell Chemical Biology

Structural Lipids Enable the Formation of Functional Oligomers of the Eukaryotic Purine Symporter UapA

Graphical Abstract

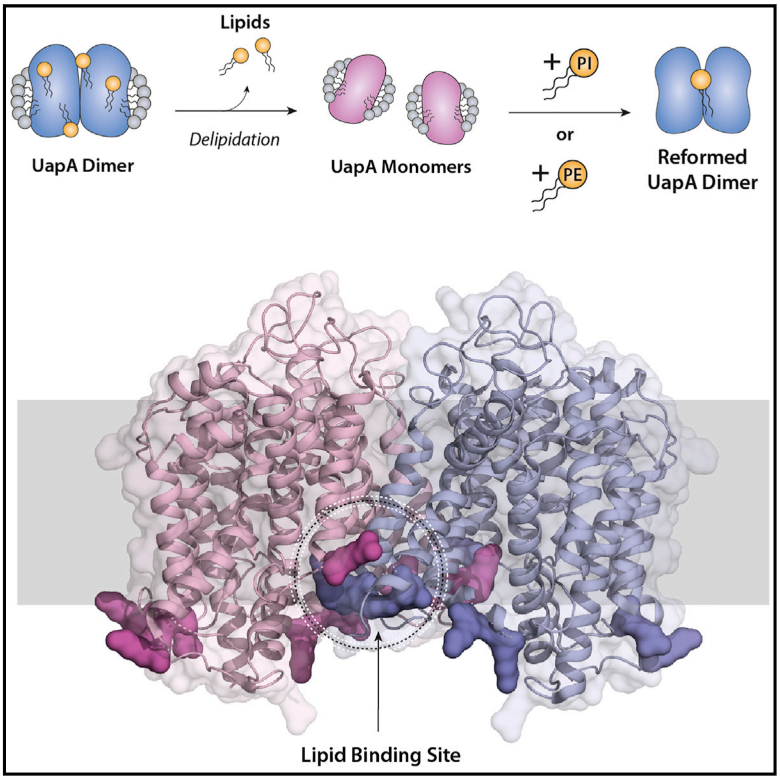

Authors

Euan Pyle, Antreas C. Kalli, Sotiris Amillis, ..., George Diallinas, Bernadette Byrne, Argyris Politis

Correspondence

b.byrne@imperial.ac.uk (B.B.), argyris.politis@kcl.ac.uk (A.P.)

In Brief

We describe the first in-depth analysis of membrane lipid interactions with a eukaryotic transporter using native mass spectrometry. We demonstrate that the binding of structural lipids is essential to maintain the stability of the functional UapA dimer in both the gas phase and *in vivo*.

Highlights

- Mass spectrometry reveals specific lipid binding to the eukaryotic transporter UapA
- Interfacial lipids stabilize the functional UapA dimer
- MD simulations reveal the lipid binding sites
- Mutagenesis of a lipid binding site disrupts UapA dimerization and function *in vivo*

Pyle et al., 2018, Cell Chemical Biology *25*, 840–848
July 19, 2018 © 2018 The Authors. Published by Elsevier Ltd.
https://doi.org/10.1016/j.chembiol.2018.03.011

CellPress

Cell Chemical Biology
Article

Structural Lipids Enable the Formation of Functional Oligomers of the Eukaryotic Purine Symporter UapA

Euan Pyle,[1,2] Antreas C. Kalli,[3] Sotiris Amillis,[4] Zoe Hall,[5] Andy M. Lau,[2] Aylin C. Hanyaloglu,[6] George Diallinas,[4] Bernadette Byrne,[1,*] and Argyris Politis[2,7,*]
[1]Department of Life Sciences, Imperial College London, London SW7 2AZ, UK
[2]Department of Chemistry, King's College London, London SE1 1DB, UK
[3]Leeds Institute of Cancer & Pathology and Astbury Centre for Structural Molecular Biology, University of Leeds, Leeds LS2 9JT, UK
[4]Department of Biology, National and Kapodistrian University of Athens, Panepistimioupolis, 15781 Athens, Greece
[5]Department of Biochemistry, University of Cambridge, Cambridge CB2 1GA, UK
[6]Institute of Reproductive and Developmental Biology, Department of Surgery and Cancer, Imperial College London, London W12 0NN, UK
[7]Lead Contact
*Correspondence: b.byrne@imperial.ac.uk (B.B.), argyris.politis@kcl.ac.uk (A.P.)
https://doi.org/10.1016/j.chembiol.2018.03.011

SUMMARY

The role of membrane lipids in modulating eukaryotic transporter assembly and function remains unclear. We investigated the effect of membrane lipids in the structure and transport activity of the purine transporter UapA from *Aspergillus nidulans*. We found that UapA exists mainly as a dimer and that two lipid molecules bind per UapA dimer. We identified three phospholipid classes that co-purified with UapA: phosphatidylcholine, phosphatidylethanolamine (PE), and phosphatidylinositol (PI). UapA delipidation caused dissociation of the dimer into monomers. Subsequent addition of PI or PE rescued the UapA dimer and allowed recovery of bound lipids, suggesting a central role of these lipids in stabilizing the dimer. Molecular dynamics simulations predicted a lipid binding site near the UapA dimer interface. Mutational analyses established that lipid binding at this site is essential for formation of functional UapA dimers. We propose that structural lipids have a central role in the formation of functional, dimeric UapA.

INTRODUCTION

Cellular membranes play key roles in determining the structure and function of membrane proteins (Opekarova and Tanner, 2003). Membranes are highly fluid and asymmetrical structures that provide distinct physical environments for the associated proteins (Engelman, 2005). The biophysical properties of the membrane, such as lateral and transverse pressures caused by membrane curvature and lipid packing, directly affect membrane protein folding, structure, and function (Booth and Curnow, 2009; Marsh, 1996; van den Brink-van der Laan et al., 2004). Furthermore, both specific and non-specific protein-lipid interactions can affect transporter conformation, stability, and oligomerization (Gupta et al., 2017; Koshy and Ziegler, 2015; Laganowsky et al., 2014; Martens et al., 2016). For example, specific lipid binding to subunit-subunit interfaces can modulate BetP oligomerization (Koshy et al., 2013). In contrast, bulk or annular lipid interactions can provide structural support to facilitate conformational changes in the transporter NapA by stabilizing the position of a static gate domain while allowing the movement of dynamic core domain to accommodate an elevator-like mechanism (Landreh et al., 2017). Therefore, it is essential to characterize protein-lipid interactions and the relationship between proteins and the cell membrane to fully understand the structure and function of membrane proteins *in vivo*.

Traditional structural biology methods, such as cryoelectron microscopy and X-ray crystallography, have offered invaluable information regarding membrane protein structure and function; however, even with these high-resolution methods capturing protein-lipid interactions remains a challenge (Peng et al., 2014). While there has been a marked increase in the number of membrane protein structures with resolved lipids in recent years, only a select few studies have established the structure-function relationship of protein-lipid interactions (Koshy et al., 2013; Mehmood et al., 2016; Norimatsu et al., 2017). Mass spectrometry (MS) of intact protein complexes has emerged as a key method for detecting, identifying, and characterizing protein-lipid interactions (Gupta et al., 2017; Henrich et al., 2017; Laganowsky et al., 2013; Landreh et al., 2017; Mehmood et al., 2016; Reading et al., 2015; Skruzny et al., 2015; Zhou et al., 2011). Native MS employs nano-electrospray ionization as a "soft" ionization technique that preserves the structure, oligomerization, and ligand-lipid interactions of protein complexes (Ahdash et al., 2017; Ashcroft, 2005; Hernandez and Robinson, 2007). During native MS of intact membrane proteins, detergent micelles are removed through collisional activation with inert gas molecules. Careful tuning of the instrument parameters ensures well-resolved spectra, while maintaining the overall protein fold as well as interactions with other proteins, ligands, and/or lipids. Consequently, native MS has emerged as a powerful tool for examining the role of lipid binding on the stability of membrane

 © 2018 The Authors. Published by Elsevier Ltd.
This is an open access article under the CC BY license (http://creativecommons.org/licenses/by/4.0/).

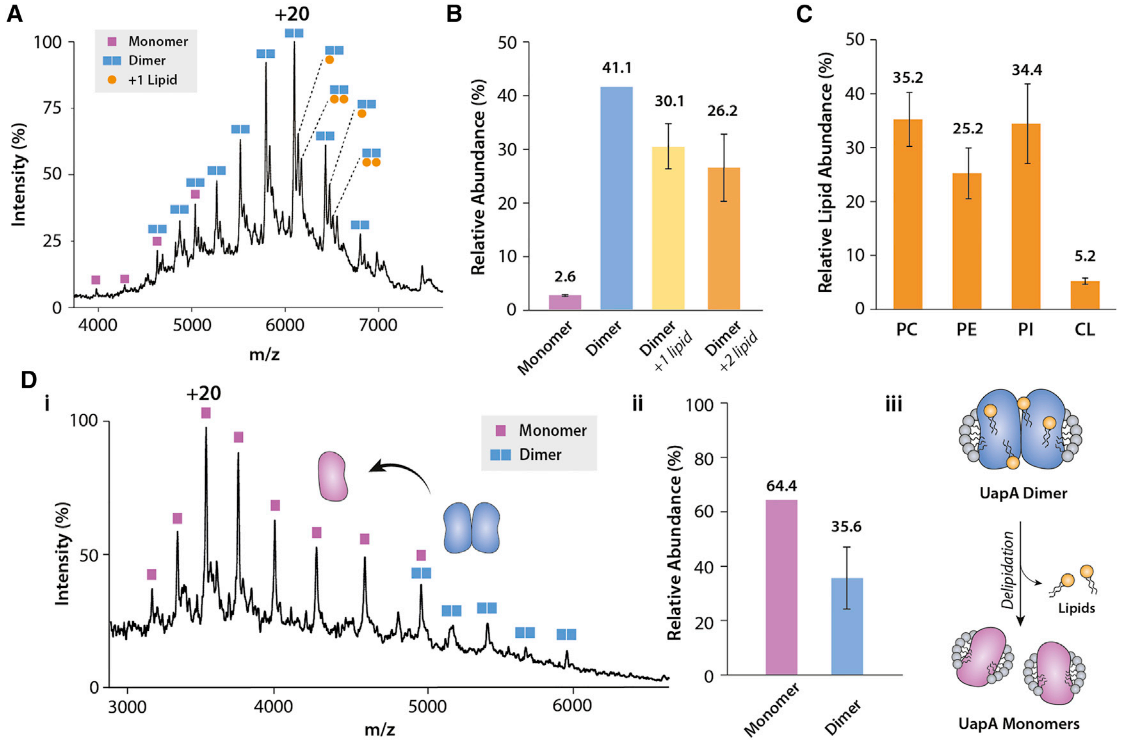

Figure 1. Bound Lipids Affect the Oligomerization of UapAG411V$_{\Delta 1\text{-}11}$

(A and B) Mass spectrum of UapAG411V$_{\Delta 1\text{-}11}$ (A) highlighting the presence of both monomer and dimer species and lipid binding to the dimer. (B) Relative abundance of the different forms of UapAG411V$_{\Delta 1\text{-}11}$ identified by native MS.

(C) Relative abundance of each lipid class identified by LC-MS and LC-MS/MS from the lipid extract of a purified UapAG411V$_{\Delta 1\text{-}11}$ sample. PC, phosphatidylcholine; PE, phosphatidylethanolamine; PI, phosphatidylinositol; CL, cardiolipin.

(D) (i) Mass spectrum showing the effect of delipidation on the oligomerization of UapAG411V$_{\Delta 1\text{-}11}$. (ii) The relative abundance of monomer and dimer in the delipidated sample. (iii) Schematic summarizing the effects of delipidation. Removal of lipid causes dissociation of the UapAG411V$_{\Delta 1\text{-}11}$ dimer into monomers. The relative abundance of each oligomeric species in (B), (C), and (Dii) was quantified using UniDec software (Marty et al., 2015). The mass spectra are representative of three independent experiments carried out under identical conditions. The relative abundance data are the average ± SD (n = 3) and the average values are given above the bars on each chart.

protein oligomers (Gupta et al., 2017; Reading et al., 2015; Wang et al., 2010).

Recent studies using native MS have demonstrated that protein-lipid interactions play a crucial role in stabilizing the dimer form of prokaryotic transporters (Gupta et al., 2017; Henrich et al., 2017). However, our understanding of the role of lipids in maintaining the quaternary structure of eukaryotic transporters remains limited, mainly due to the relatively poor stability of these proteins in non-native environments. Here, we used native MS to study UapA, a eukaryotic transporter from *Aspergillus nidulans* belonging to the nucleobase ascorbate transporter (NAT) family of metabolite importers. UapA is responsible for H^+-dependent uptake of the purines xanthine and uric acid. The high-resolution structure of a transport inactive, conformationally locked mutant of UapA (G411V$_{\Delta 1\text{-}11}$) was recently solved, revealing that UapA is formed from two domains, the core domain and gate domain, and is likely to transport via an elevator mechanism (Alguel et al., 2016). The structure also showed that UapA is a homodimer confirming earlier biochemical studies (Martzoukou et al., 2015). Analysis of the structure in combination with mutagenesis data demonstrated that dimer formation was essential for function (Alguel et al., 2016). The structure showed that the extensive (~6,000 A^2) dimer interface was mainly mediated by hydrophobic interactions and displayed no electron density consistent with lipid binding (Alguel et al., 2016). We combined native MS with molecular dynamics (MD), mutagenesis, and functional analyses to establish that phosphatidylinositol (PI) and phosphatidylethanolamine (PE) are closely associated with UapA and play a crucial role in stabilizing the functional UapA dimer.

RESULTS

Protein-Lipid Interactions Stabilize the UapA Dimer

We began by subjecting a purified thermostabilized, conformationally locked, inward-facing mutant of UapA (G411V$_{\Delta 1\text{-}11}$) (MW = 60,859.59 Da) (STAR Methods and Figure S1A) to ion-mobility (IM)-MS (Leung et al., 2013). IM-MS reports the molecular shape of biomolecules by determining their rotationally averaged collisional cross-section (CCS) (Jenner et al., 2011; Jurneczko and Barran, 2011; Konijnenberg et al., 2014; Michaelevski et al., 2010; Ruotolo et al., 2008). We found the experimental CCS of UapAG411V$_{\Delta 1\text{-}11}$ (6,117 Å) to be in agreement with the theoretical CCS of the native-like state, calculated from the

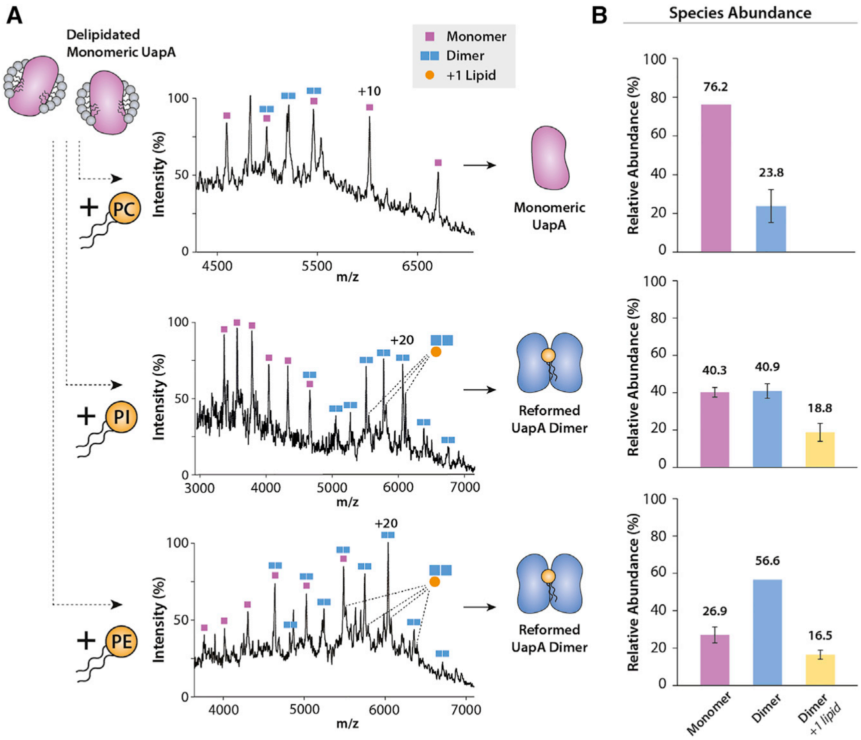

Figure 2. Addition of Lipids to Delipidated UapAG411V$_{\Delta1\text{-}11}$ Reforms the Dimer

(A) Mass spectra showing the effects on oligomerization of adding PC (34:1, upper), PI (34:1, middle), and PE (34:1, lower) to delipidated UapAG411V$_{\Delta1\text{-}11}$. Lipid binding peaks are highlighted. Lipids were added to delipidated UapAG411V$_{\Delta1\text{-}11}$ at a ratio of 100:1 lipid/UapA.

(B) Relative abundances of monomer and dimer species in the presence of PC (upper), PI (middle), and PE (lower) were quantified using UniDec software (Marty et al., 2015). The mass spectra are representative of three independent experiments carried out under identical conditions. The relative abundance data are the average ± SD (n = 3) and the average values are given above each bar.

crystal structure of UapAG411V$_{\Delta1\text{-}11}$ (6,230 Å) (PDB: 5I6C), indicating that the protein remains folded in the gas phase (Figure S1B).

Next, to investigate the oligomeric states of UapA, we performed native MS on UapAG411V$_{\Delta1\text{-}11}$. Well-resolved spectra showed that the protein exists both as a monomer and a dimer (Figures 1A and 1B). The relative abundance of monomer/dimer was ~5:95, suggesting a strong dimer interface (Yefremova et al., 2017). The spectra also revealed the binding of two adduct molecules (measured mass: 775 ± 50 Da) to the UapAG411V$_{\Delta1\text{-}11}$ dimer. We attributed this to the presence of bound phospholipids; however, the resolution of the mass spectra was insufficient to allow identification of the lipid species. We therefore extracted the lipids from the purified protein and analyzed the lipid extract by liquid chromatography-MS (LC-MS) and tandem MS (LC-MS/MS) experiments. We identified phosphatidylcholine (PC), PE, and PI as the major lipid classes co-purifying with UapAG411V$_{\Delta1\text{-}11}$ (Figures 1C and S2; Table S1). We verified the presence of the lipids identified by LC-MS in the UapAG411V$_{\Delta1\text{-}11}$ sample by negative-ion native MS (Figure S1C). PE, PC, and PI are all abundant species in the plasma membranes of both the expression host (*Saccharomyces cerevisiae*) and the native host (*A. nidulans*). Consequently, their interactions with UapA are likely to be physiologically relevant (Birch et al., 1998).

To study the effect of lipids in the formation of UapA dimers, we delipidated UapAG411V$_{\Delta1\text{-}11}$ and subjected it to native MS. Strikingly, our results showed that the delipidated protein is present primarily in the monomeric form (Figure 1D), suggesting that the lipids are important in maintaining the physiological dimer, as previously seen for prokaryotic transporters (Gupta et al., 2017). Loss of peaks in the ~750 *m/z* range of the native MS spectra further confirmed almost total removal of lipid from the samples (Figure S3A). To explore the effect of the different lipids identified in lipidomics on UapA dimerization, we added PI, PE, or PC individually, or in combination, into delipidated UapAG411V$_{\Delta1\text{-}11}$ at a ratio of 1:100 protein/lipid (Figure 2). Addition of either PE or PI restored the UapA dimer with both lipids displaying similar levels of dimer recovery, yielding 73.1% and 59.7% dimer, respectively (Figure 2). The addition of an equimolar mixture of PI and PE to delipidated UapAG411V$_{\Delta1\text{-}11}$ was more efficient at dimer recovery, yielding 81.5% dimer (Figure S3C). This implies that the effects of the two lipids are additive. As a negative control we also titrated phosphatidylglycerol (PG), a lipid not usually found in eukaryotic plasma membranes, into delipidated UapAG411V$_{\Delta1\text{-}11}$ (Opekarova and Tanner, 2003). PG did not induce any significant changes in the oligomerization of delipidated UapAG411V$_{\Delta1\text{-}11}$ (Figure S3B).

In contrast to PI and PE, the addition of PC to delipidated UapAG411V$_{\Delta1\text{-}11}$ failed to mediate the reformation of the UapA dimer. Indeed, adding PC actively induced dissociation of non-delipidated UapAG411V$_{\Delta1\text{-}11}$ (Figure S3D). Surprisingly, the presence of PC inhibited the dimer-stabilizing effects of PI and PE when mixtures of the three lipids were added to delipidated UapAG411V$_{\Delta1\text{-}11}$ (Figure S3C). Taken together, these data suggest that the dimer-stabilizing effects are specific to PI and PE lipids.

Next, we investigated the effects of lipid binding on the functionally active wild-type (WT) UapA (MW = 61,972.71 Da). We found the relative abundance of monomer/dimer to be approximately 50:50. This is likely to be due to the reduced stability of WT UapA compared with UapAG411V$_{\Delta1\text{-}11}$ (Leung et al., 2013) (Figure S4). Delipidation of the WT protein resulted in almost complete loss of the dimer form; this could be partially recovered by addition of PI (Figure S4).

MD Simulations Predict Lipid Binding Sites at the Dimer Interface

MD simulations were performed to predict likely lipid binding sites. We carried out 5-μs simulations using a model of WT

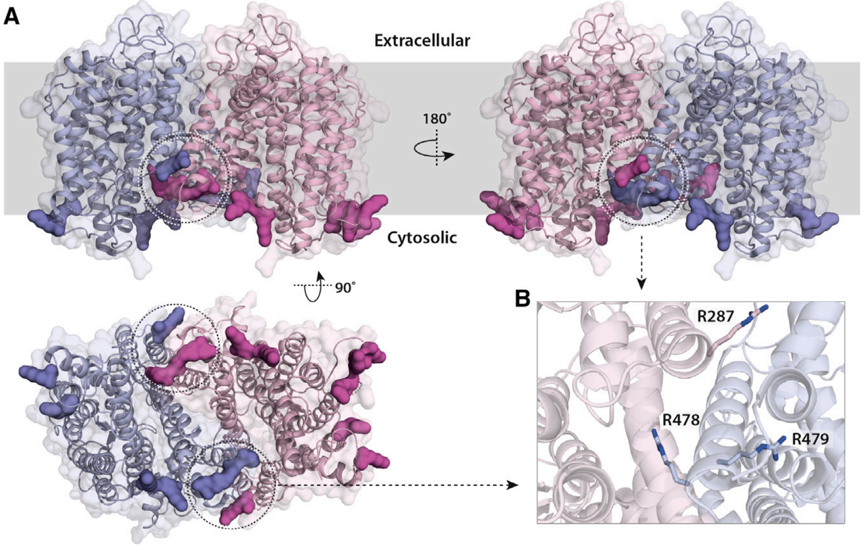

Figure 3. MD Simulations Predict a Lipid Binding Site at the Dimer Interface of UapA

The simulations were carried out in a symmetric bilayer of PC/PE/PI 40:25:35.

Cartoon representation of UapA (A) with one monomer colored blue and the other colored pink. The protein is shown from both sides looking through the membrane (upper panel) and from the intracellular side of the membrane (lower panel). Residues with a normalized contact probability with PI higher than 0.8 over the course of the simulation (Figure S5B) are shown in a space-filling representation in dark blue or dark pink. The region highlighted in the dotted circle indicates those residues predicted to form a lipid binding site at the dimer interface of UapA and is shown in close-up view in (B), where individual amino acid residues are shown in stick representation and labeled with the residue number.

UapA based on the crystal structure of UapAG411V$_{\Delta 1\text{-}11}$ (PDB: 5I6C). The structure was centered and embedded into a symmetric membrane containing PC (40%), PE (25%), and PI (35%), reflecting the lipid composition revealed via lipidomics. Due to the long simulation time, we coarse-grained the molecules and used the Martini force field (Marrink and Tieleman, 2013). In these simulations, the inward-facing conformation of UapA was locked using an elastic network model. We analyzed our simulations to predict any likely lipid binding sites near the dimer interface that may play a role in stabilizing the dimer. Our MD simulations predicted that PC and PE interact with the same residues and that PI interacts with UapA residues with greater frequency than either PE or PC (Figure S5A). No significant enrichment of PC or PE in the annular layer around UapA was observed. However, a number of positively charged Lys and Arg residues located principally on the intracellular side of the protein were predicted to be involved in PI binding (Figure 3A). Of particular interest was one clear PI binding site at the UapA dimer interface, comprising residues Arg287 (at the cytoplasmic end of transmembrane 7 [TM7]), Arg478, and Arg479 (at the cytoplasmic end of TM13) (Figure 3B).

Mutations to the Putative Lipid Binding Site Cause Loss of UapA Function *In Vivo*

To explore the role of the putative PI binding site in the structure and function of the UapA dimer, we generated a range of single, double, and triple mutants of the residues (R287A, R478A, R479A, R478A/R479A, R287A/R478A/R479A) with C-terminal GFP tags. We individually transformed these constructs into a strain of *A. nidulans* (*uapAΔ uapCΔ azgAΔ pabaA1 argB2*) lacking all endogenous nucleobase transport systems. Transformants expressing the individual UapA mutants were tested for their capacity to grow using uric acid or xanthine as the sole nitrogen source. At 37°C, only the triple R287A/R478A/R479A mutant showed markedly impaired growth on either uric acid- or xanthine-supplemented media, indicating a loss of UapA transport activity (Figure 4A). In contrast, at 25°C, closer to the physiological temperature of *A. nidulans*, both the triple R287A/R478A/R479A and double R478A/R479A mutants were unable to grow on either uric acid or xanthine. The single R479A mutant also showed reduced growth on both substrates (Figure 4A).

Fluorescence microscopy carried out at 25°C showed that all of the GFP-tagged UapA mutants localized effectively to the plasma membrane, confirming that loss of transport activity in the mutants is not a result of impaired UapA trafficking (Figure 4B). Further analysis using [^{3}H]xanthine uptake assays (Figures 4C and 4D) confirmed the results from the growth assays and revealed close to WT substrate binding affinity for all mutants. This implies that the R478A/R479A and R287A/R478A/R479A mutations affect substrate transport rather than substrate binding. We have previously shown that dimer formation is critical for UapA function (Alguel et al., 2016), so in order to examine the oligomeric status of the UapA mutants we carried out bimolecular fluorescence complementation (BiFC) analysis using a split YFP system (Martzoukou et al., 2015). This system uses two copies of the individual mutants co-expressed as fusions with either the N- or C-terminal domains of YFP. In this case, fluorescence is observed upon dimerization of copies of UapA tagged with the different YFP domains. Although the R287A/R478A/R479A mutant (RRR/A) was found to traffic to the membrane (Figure 4B), it was much less efficient at reconstituting the YFP (Figure 5A). This suggested that the RRR/A mutant was principally trafficking as a monomer.

To assess the precise oligomeric status of the RRR/A mutant, we introduced the additional G411V mutation and 11-residue N-terminal truncation to generate the construct UapA RRR/A + G411V$_{\Delta 1\text{-}11}$. Native MS of the purified UapA RRR/A + G411V$_{\Delta 1\text{-}11}$ (MW = 60,604.26 Da) (Figure S1A) showed a dramatic reduction in the abundance of UapA dimer compared with UapAG411V$_{\Delta 1\text{-}11}$ (Figures 5C and 5D). In addition, there was almost complete loss of lipid binding. These data strongly suggest that the disruption of the putative lipid binding site, and consequent loss of UapA activity *in vivo*, is due to the reduction of UapA-lipid interactions that play a key role in stabilizing the functional dimer form. There is evidence of minor lipid

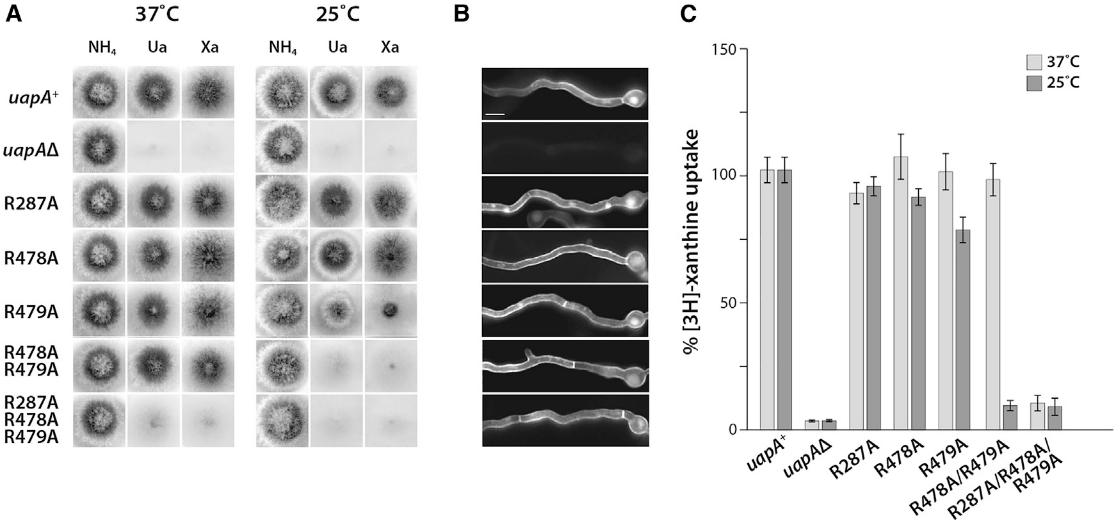

D

	uapA+	R287A	R478A	R479A	R478A R479A	R287A/R478A/ R479A
K_m (µM)	5.5 ± 1.3	13.4 ± 2.8	3.4 ± 0.9	4.5 ± 0.6	3.8 ± 1.4	1.3 ± 0.4

Figure 4. Mutation of the Lipid Binding Site at the UapA Dimer Interface Results in Loss of Transport Function

The constructs were transformed into the *A. nidulans* strain (*uapAΔ uapCΔ azgAΔ pabaA1 argB2*). uapAΔ indicates the original recipient strain as a negative control.

(A) Growth tests of *A. nidulans* strains in minimal media supplemented with either ammonium (NH_4^+), uric acid (Ua), or xanthine (Xa) as the nitrogen source. Growth was assessed at 37°C and 25°C.

(B) Inverted fluorescence microscopy images showing localization of the GFP-tagged UapA constructs. All mutants display normal sorting to the membrane. Scale bar corresponds to 5 µm.

(C) [^{3}H]Xanthine uptake assays, with rate of WT UapA uptake defined as 100%.

(D) Table of K_M values of the UapA mutants. The fungal growth and localization data are representative of three independent experiments carried out under identical conditions. The K_M and fluorescence quantification data are the average ± SD, n = 3.

binding peaks to the UapA RRR/A + G411V$_{\Delta 1-11}$ construct and limited dimer reformation after the addition of PI at a ratio of 1:100 protein/lipid, although at a much lower level than that seen for UapA G411V$_{\Delta 1-11}$. It is possible that some of the lipid binding we observe is due to PI binding to the alternative binding sites in UapA predicted by our MD simulations. Furthermore, it is also possible that lipid can still associate to the altered binding site at the dimer interface but with markedly reduced affinity, explaining why the protein purifies as a monomer.

DISCUSSION

Recent years have seen a growing understanding of the key roles played by membrane lipids in the structure and function of membrane proteins; however, as yet there has been no in-depth study of the effects of lipids on eukaryotic transporters. Here, we explore the roles of lipids in maintaining the functional dimeric form of the eukaryotic UapA transporter. We combine native MS with MD simulations and validate the physiological relevance of their findings using mutagenesis and *in vivo* functional characterization. Principally, we used the UapAG411V$_{\Delta 1-11}$ construct, showing that it primarily exists as a dimer in the gas phase and that removal of tightly bound lipids causes dissociation into a monomer. The dimer can be recovered by addition of PI or PE. Importantly, we obtained similar results for WT UapA, although the spectra acquired for this protein are of reduced quality compared with the UapAG411V$_{\Delta 1-11}$ construct due to the lower stability of the WT protein. However, this gave us confidence that further studies using UapAG411V$_{\Delta 1-11}$ were representative of the native protein.

As mutagenesis of the putative lipid binding site abolished lipid binding, we speculate that PI and PE have defined binding sites and therefore act as so-called structural lipids, forming integral interactions with UapA (Bechara and Robinson, 2015). The dimer interface of UapA has a substantial surface area of ~6,000 A^2 (Alguel et al., 2016), which should render the dimer very stable; thus, the dissociation of the dimer into the monomeric form upon delipidation may be a result of the comparatively harsh treatment of the molecule rather than a direct effect of associated lipids. However, the fact that the dimer can be reformed by the addition of PI, PE, or a combination of both lipids is strongly suggestive that these lipids have a specific stabilizing effect on the oligomeric form of the transporter. There does not seem to be an absolute requirement for either PI or PE, as individually both can recover dimer from delipidated protein to a similar extent. Interestingly, the addition of both PI and PE was more effective at dimer stabilization than the addition of PI or PE individually, suggesting the effects of these two lipids is additive. MD simulations predict that these lipids bind different regions of the protein. Together, this suggests that the dimer can be further stabilized by interactions at an alternative lipid

CellPress

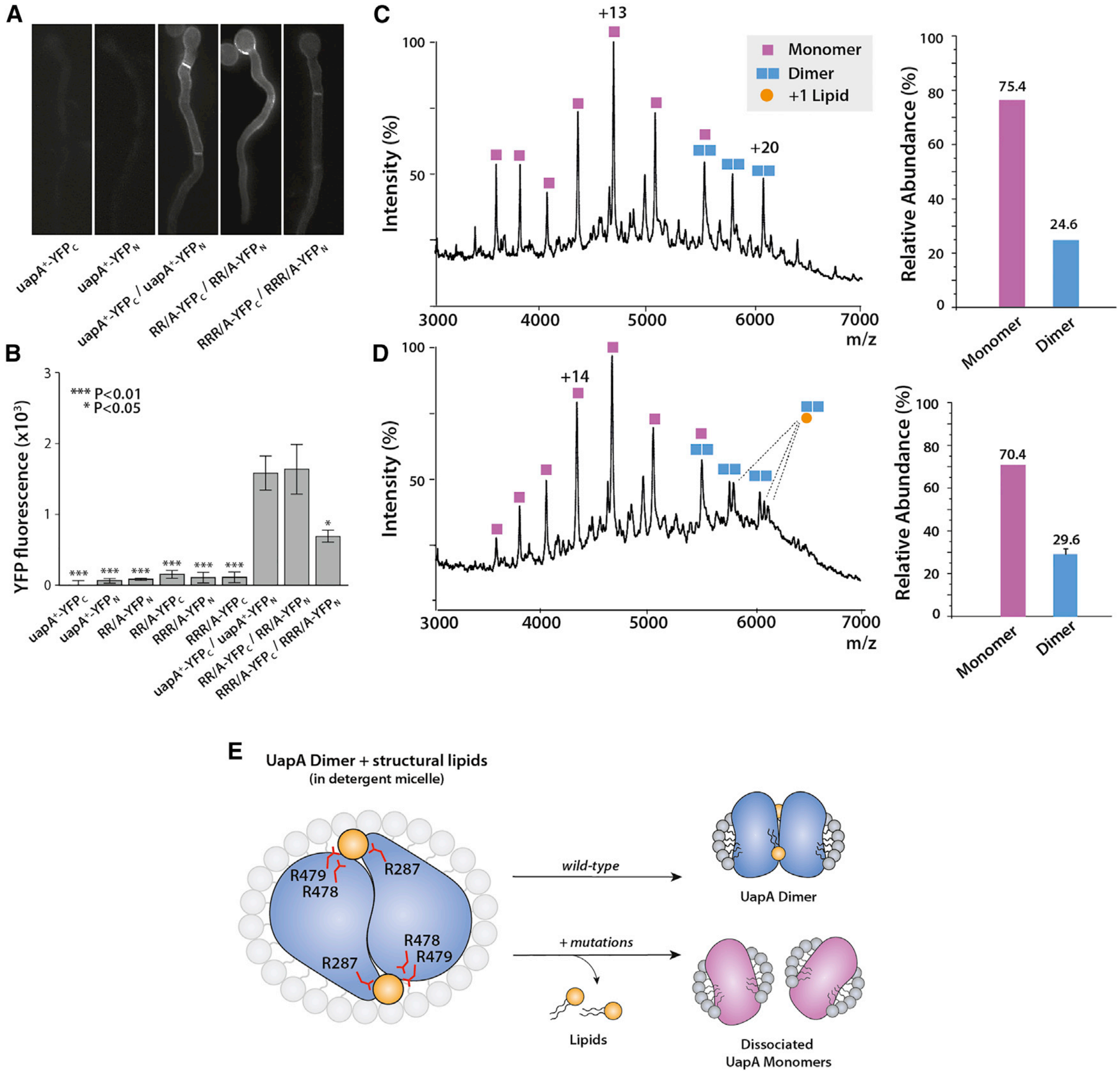

Figure 5. The R287A/R478A/R479A UapA Mutant Reduces Dimer Formation

(A) Bimolecular complementation (BiFC) analysis of the R478A/R479A (RR/A) UapA mutant and the R287A/R478A/R479A (RRR/A) UapA mutant. Mutant constructs tagged with the individual YFP domains were co-expressed in *A. nidulans*. Upon UapA dimerization the YFP is reconstituted. YFP fluorescence was measured by epifluorescence inverted microscopy. WT UapA (uapA^{+}) expressed individually with either the C-terminal domain of YFP (YFPC) or the N-terminal domain of YFP (YFPN) is the negative control.

(B) Relative quantification of plasma membrane fluorescence intensity of mutants compared with WT UapA +/− YFPC/UapA +/− YFPN.

(C) (Left) Mass spectrum of UapA RRR/A with a G411V mutation and 11-residue N-terminal truncation. (Right) Relative abundances of each oligomer of UapA RRR/A + G411V$_{\Delta 1\text{-}11}$.

(D) (Left) Mass spectrum of UapA RRR/A + G411V$_{\Delta 1\text{-}11}$ with PI (34:1) added at a ratio of 1:100 protein/lipid. (Right) Relative abundances of each oligomer of RRR/A + G411V$_{\Delta 1\text{-}11}$ with PI (34:1) added at a ratio of 1:100 protein/lipid. See Figure 1A for the relative abundances of each oligomer of UapAG411V$_{D1\text{-}11}$ under identical conditions. The relative abundance of each species was quantified using UniDec software (Marty et al., 2015). The mass spectrum is representative of three independent experiments carried out under identical conditions. The relative abundance data are the average ± SD, n = 3.

(E) Schematic showing the effect of the R287/R479/R478 mutations on lipid stabilization of the UapA dimer. Mutations of R287A/R478A/R479A abolish lipid binding capability, resulting in the dissociation of UapA into monomers.

binding site. It is also possible that PE binding induces positive allosteric modulation of PI binding; such effects have been reported previously for AmtB (Patrick et al., 2018). Further research will be required to confirm the location of alternative dimer-stabilizing lipid binding sites.

Interestingly, MD simulations predicted that most specific lipid binding sites are found within regions of the protein located on the intracellular side of the membrane and are therefore in contact with lipids in the inner membrane leaflet. Similar predictions have been recently made for NapA (Landreh et al., 2017) and CitS (Wöhlert et al., 2015) transporters. A high-resolution crystal structure of BetP also revealed that asymmetry of lipids is important in mediating oligomer formation with a preference for lipids in the inner leaflet (Koshy et al., 2013).

Of the three residues comprising the putative lipid binding site at the UapA dimer interface, only R479 is highly conserved among the eukaryotic NAT proteins. Interestingly, the R479A mutation was the only single substitution that had detectable effects on the *A. nidulans* growth rate. A loss of function observed for the R478A/R479A double mutant at the physiological temperature for *A. nidulans*, 25°C, and for the R287A/R478A/R479A triple mutant at both 25°C and 37°C was not due to either a loss of correct trafficking to the membrane or impaired substrate binding. The apparent cryosensitivity of the single (R479A) and double (R478A/R479A) mutants may be due to a change in the *A. nidulans* lipid composition at the lower temperature. Such temperature-dependent lipid composition changes have been reported for *S. cerevisiae* (Ejsing et al., 2009).

Native MS analysis of the purified triple mutant in the G411V$_{\Delta 1\text{-}11}$ background indicated that this construct is almost exclusively monomeric. Given that the functional form of UapA is the dimer, we reason that the lack of transport function in this protein is due to its inability to bind lipids that promote dimerization. However, it is important to note that both native MS and the BiFC assay indicate that dimer does form in the absence of lipid binding, although to a much lower extent than when the lipid binding site at the dimer interface is intact. However, this form of the protein has lost virtually all transport activity. This indicates that lipid binding is not an absolute requirement for dimer formation but is essential for formation of the functional dimer, which suggests that lipid binding could form part of the regulatory mechanism of UapA. This hypothesis could form the basis of future investigations into the nature of UapA-lipid interactions.

The MD simulations predicted that lipids could also bind to the outermost, membrane-facing regions of the core domains of the UapA dimer (see Figure S1D for domain organization of UapA). Lipid binding to this region has been predicted for NhaA and suggested to be involved in stabilizing the core domain during the conformational transitions required for transport by the elevator mechanism (Landreh et al., 2017). However, in the case of UapA, removal of the putative lipid binding site at the dimer interface caused almost total loss of lipid binding as revealed by native MS (Figure 5C). This indicates that binding to the core domain may not be a feature of UapA-lipid interactions. Further research is required to confirm this.

In conclusion, we have performed the first detailed analysis of the role of lipid binding to a eukaryotic transporter UapA, and have shown that specific structural lipids are critical for maintaining the protein in a functional dimeric state. Overall, the combination of approaches used here has clear potential to allow investigation of the function of lipid binding to a range of different membrane proteins.

SIGNIFICANCE

Lipids play an important role in the stability and function of membrane transporters. It has been proposed that the binding of lipids to specific sites on membrane proteins is integral to both protein structure and function. Here, we have characterized structural lipids associated with a eukaryotic transporter, UapA, using native mass spectrometry and *in vivo* functional analysis. We propose that structural lipids stabilize the dimer interface of UapA and are essential for transport activity. These findings expand our understanding of eukaryotic protein-lipid interactions and have direct implications for the molecular mechanism of UapA transport.

STAR★METHODS

Detailed methods are provided in the online version of this paper and include the following:

- KEY RESOURCES TABLE
- CONTACT FOR REAGENT AND RESOURCE SHARING
- METHOD DETAILS
  - Expression of the UapA Constructs and Membrane Preparation
  - Purification of UapA
  - Native MS
  - Lipid Titrations
  - IM-MS
  - LC-MS
  - Coarse-grained Molecular Dynamics (CG-MD) Simulations
  - *A. nidulans* Growth Conditions, UapA Localisation, Bifluorescence Complementation Assays and Xanthine Transport Assays
- QUANTIFICATION AND STATISTICAL ANALYSIS

SUPPLEMENTAL INFORMATION

Supplemental Information includes five figures and one table and can be found with this article online at https://doi.org/10.1016/j.chembiol.2018.03.011.

ACKNOWLEDGMENTS

The research was funded by the Wellcome Trust (109854/Z/15/Z to A.P.) and the Biotechnology and Biological Sciences Research Council grant BB/K017292/1 to B.B. Work in the laboratory of G.D. was supported by a Stavros S. Niarchos Foundation for Charity grant. E.P. is the recipient of an Imperial College London Institute of Chemical Biology EPSRC CDT studentship awarded to B.B., A.P., and A.C.H. We would like to thank Drs, Eamonn Reading and Chloé Martens for their help in native MS experimental design, and Dr, Yilmaz Alguel for guidance in protein expression and purification. We thank Professor Julian Griffin for access to Orbitrap MS instrumentation. The MD simulations were undertaken on ARC2 and MARC1, part of the High Performance Computing and Leeds Institute for Data Analytics (LIDA) facilities at the University of Leeds, UK.

AUTHOR CONTRIBUTIONS

A.P. and B.B. designed the project; E.P. expressed and purified the protein; Z.H. carried out lipidomics experiments; E.P. analyzed the protein by native MS and IM-MS; A.C.K. performed MD simulations; S.A. and G.D. carried out the mutagenesis functional analysis and localization studies of the protein; A.M.L. and A.C.H. analyzed the data; E.P., B.B., and A.P. wrote the manuscript with contributions from all authors.

DECLARATION OF INTERESTS

The authors declare no competing interests.

Received: December 8, 2017
Revised: February 27, 2018
Accepted: March 22, 2018
Published: April 19, 2018

REFERENCES

Ahdash, Z., Lau, A.M., Byrne, R.T., Lammens, K., Stüetzer, A., Urlaub, H., Booth, P.J., Reading, E., Hopfner, K.P., and Politis, A. (2017). Mechanistic insight into the assembly of the HerA-NurA helicase-nuclease DNA end resection complex. Nucleic Acids Res. *45*, 14.

Alguel, Y., Amillis, S., Leung, J., Lambrinidis, G., Capaldi, S., Scull, N.J., Craven, G., Iwata, S., Armstrong, A., Mikros, E., et al. (2016). Structure of eukaryotic purine/H(+) symporter UapA suggests a role for homodimerization in transport activity. Nat. Commun. *7*, 11336.

Allison, T.M., Reading, E., Liko, I., Baldwin, A.J., Laganowsky, A., and Robinson, C.V. (2015). Quantifying the stabilizing effects of protein-ligand interactions in the gas phase. Nat. Commun. *6*, 8551.

Ashcroft, A.E. (2005). Recent developments in electrospray ionisation mass spectrometry: noncovalently bound protein complexes. Nat. Prod. Rep. *22*, 452–464.

Bechara, C., and Robinson, C.V. (2015). Different modes of lipid binding to membrane proteins probed by mass spectrometry. J. Am. Chem. Soc. *137*, 5240–5247.

Birch, M., Drucker, D.B., Riba, I., Gaskell, S.J., and Denning, D.W. (1998). Polar lipids of *Aspergillus fumigatus*, *A. niger*, *A. nidulans*, *A. flavus* and *A. terreus*. Med. Mycol. *36*, 127–134.

Booth, P.J., and Curnow, P. (2009). Folding scene investigation: membrane proteins. Curr. Opin. Struct. Biol. *19*, 8–13.

Bush, M.F., Hall, Z., Giles, K., Hoyes, J., Robinson, C.V., and Ruotolo, B.T. (2010). Collision cross sections of proteins and their complexes: a calibration framework and database for gas-phase structural biology. Anal. Chem. *82*, 9557–9565.

Bussi, G., Donadio, D., and Parrinello, M. (2007). Canonical sampling through velocity rescaling. J. Chem. Phys. *126*, 014101.

Clarke, D.J., and Campopiano, D.J. (2015). Desalting large protein complexes during native electrospray mass spectrometry by addition of amino acids to the working solution. Analyst *140*, 2679–2686.

David Van Der, S., Erik, L., Berk, H., Gerrit, G., Alan, E.M., and Herman, J.C.B. (2005). GROMACS: fast, flexible, and free. J. Comput. Chem. *26*, 1701–1718.

de Jong, D.H., Singh, G., Bennett, W.F.D., Arnarez, C., Wassenaar, T.A., Schäfer, L.V., Periole, X., Tieleman, D.P., and Marrink, S.J. (2013). Improved parameters for the Martini coarse-grained protein force field. J. Chem. Theor. Comput. *9*, 687–697.

Ejsing, C.S., Sampaio, J.L., Surendranath, V., Duchoslav, E., Ekroos, K., Klemm, R.W., Simons, K., and Shevchenko, A. (2009). Global analysis of the yeast lipidome by quantitative shotgun mass spectrometry. Proc. Natl. Acad. Sci. USA *106*, 2136–2141.

Engelman, D.M. (2005). Membranes are more mosaic than fluid. Nature *438*, 578–580.

Fahy, E., Sud, M., Cotter, D., and Subramaniam, S. (2007). LIPID MAPS online tools for lipid research. Nucleic Acids Res. *35*, W606–W612.

Fiser, A., and Šali, A. (2003). Modeller: generation and refinement of homology-based protein structure models. Methods Enzymol. *374*, 461–491.

Folch, J., Lees, M., and Sloane Stanley, G.H. (1957). A simple method for the isolation and purification of total lipides from animal tissues. J. Biol. Chem. *226*, 497–509.

Fraser, G.W. (2002). The ion detection efficiency of microchannel plates (MCPs). Int. J. Mass Spectrom. *215*, 13–30.

Gupta, K., Donlan, J.A.C., Hopper, J.T.S., Uzdavinys, P., Landreh, M., Struwe, W.B., Drew, D., Baldwin, A.J., Stansfeld, P.J., and Robinson, C.V. (2017). The role of interfacial lipids in stabilizing membrane protein oligomers. Nature *541*, 421–424.

Hall, Z., Politis, A., and Robinson, C.V. (2012). Structural modeling of heteromeric protein complexes from disassembly pathways and ion mobility-mass spectrometry. Structure *20*, 1596–1609.

Henrich, E., Peetz, O., Hein, C., Laguerre, A., Hoffmann, B., Hoffmann, J., Dotsch, V., Bernhard, F., and Morgner, N. (2017). Analyzing native membrane protein assembly in nanodiscs by combined non-covalent mass spectrometry and synthetic biology. Elife *6*, https://doi.org/10.7554/eLife.20954.

Hernandez, H., and Robinson, C.V. (2007). Determining the stoichiometry and interactions of macromolecular assemblies from mass spectrometry. Nat. Protoc. *2*, 715–726.

Jenner, M., Ellis, J., Huang, W.C., Lloyd Raven, E., Roberts, G.C., and Oldham, N.J. (2011). Detection of a protein conformational equilibrium by electrospray ionisation-ion mobility-mass spectrometry. Angew. Chem. Int. Ed. *50*, 8291–8294.

Jurneczko, E., and Barran, P.E. (2011). How useful is ion mobility mass spectrometry for structural biology? The relationship between protein crystal structures and their collision cross sections in the gas phase. Analyst *136*, 20–28.

Karachaliou, M., Amillis, S., Evangelinos, M., Kokotos, A.C., Yalelis, V., and Diallinas, G. (2013). The arrestin-like protein ArtA is essential for ubiquitination and endocytosis of the UapA transporter in response to both broad-range and specific signals. Mol. Microbiol. *88*, 301–317.

Koldsø, H., Shorthouse, D., Hélie, J., and Sansom, M.S.P. (2014). Lipid clustering correlates with membrane curvature as revealed by molecular simulations of complex lipid bilayers. PLoS Comput. Biol. *10*, e1003911.

Konijnenberg, A., Yilmaz, D., Ingólfsson, H.I., Dimitrova, A., Marrink, S.J., Li, Z., Vénien-Bryan, C., Sobott, F., and Koçer, A. (2014). Global structural changes of an ion channel during its gating are followed by ion mobility mass spectrometry. Proc. Natl. Acad. Sci. USA *111*, 17170–17175.

Koshy, C., Schweikhard, E.S., Gartner, R.M., Perez, C., Yildiz, O., and Ziegler, C. (2013). Structural evidence for functional lipid interactions in the betaine transporter BetP. EMBO J. *32*, 3096–3105.

Koshy, C., and Ziegler, C. (2015). Structural insights into functional lipid–protein interactions in secondary transporters. Biochim. Biophys. Acta *1850*, 476–487.

Kota, J., Gilstring, C.F., and Ljungdahl, P.O. (2007). Membrane chaperone Shr3 assists in folding amino acid permeases preventing precocious ERAD. J. Cell Biol. *176*, 617–628.

Koukaki, M., Giannoutsou, E., Karagouni, A., and Diallinas, G. (2003). A novel improved method for *Aspergillus nidulans* transformation. J. Microbiol. Methods *55*, 687–695.

Krypotou, E., and Diallinas, G. (2014). Transport assays in filamentous fungi: kinetic characterization of the UapC purine transporter of *Aspergillus nidulans*. Fungal Genet. Biol. *63*, 1–8.

Laganowsky, A., Reading, E., Allison, T.M., Ulmschneider, M.B., Degiacomi, M.T., Baldwin, A.J., and Robinson, C.V. (2014). Membrane proteins bind lipids selectively to modulate their structure and function. Nature *510*, 172–175.

Laganowsky, A., Reading, E., Hopper, J.T.S., and Robinson, C.V. (2013). Mass spectrometry of intact membrane protein complexes. Nat. Protoc. *8*, 639–651.

Landreh, M., Marklund, E.G., Uzdavinys, P., Degiacomi, M.T., Coincon, M., Gault, J., Gupta, K., Liko, I., Benesch, J.L., Drew, D., et al. (2017). Integrating mass spectrometry with MD simulations reveals the role of lipids in Na^+/H^+ antiporters. Nat. Commun. *8*, 13993.

Leung, J., Cameron, A.D., Diallinas, G., and Byrne, B. (2013). Stabilizing the heterologously expressed uric acid-xanthine transporter UapA from the lower eukaryote *Aspergillus nidulans*. Mol. Membr. Biol. *30*, 32–42.

Marrink, S.J., Risselada, H.J., Yefimov, S., Tieleman, D.P., and de Vries, A.H. (2007). The MARTINI force field: coarse grained model for biomolecular simulations. J. Phys. Chem. B *111*, 7812–7824.

Marrink, S.J., and Tieleman, D.P. (2013). Perspective on the Martini model. Chem. Soc. Rev. *42*, 6801–6822.

Marsh, D. (1996). Lateral pressure in membranes. Biochim. Biophys. Acta *1286*, 183–223.

Martens, C., Stein, R.A., Masureel, M., Roth, A., Mishra, S., Dawaliby, R., Konijnenberg, A., Sobott, F., Govaerts, C., and McHaourab, H.S. (2016). Lipids modulate the conformational dynamics of a secondary multidrug transporter. Nat. Struct. Mol. Biol. *23*, 744–751.

Marty, M.T., Baldwin, A.J., Marklund, E.G., Hochberg, G.K.A., Benesch, J.L.P., and Robinson, C.V. (2015). Bayesian deconvolution of mass and ion

CellPress

mobility spectra: from binary interactions to polydisperse ensembles. Anal. Chem. *87*, 4370–4376.

Martzoukou, O., Amillis, S., Zervakou, A., Christoforidis, S., and Diallinas, G. (2017). The AP-2 complex has a specialized clathrin-independent role in apical endocytosis and polar growth in fungi. Elife *6*, https://doi.org/10.7554/eLife.20083.

Martzoukou, O., Karachaliou, M., Yalelis, V., Leung, J., Byrne, B., Amillis, S., and Diallinas, G. (2015). Oligomerization of the UapA purine transporter is critical for ER-exit, plasma membrane localization and turnover. J. Mol. Biol. *427*, 2679–2696.

Mehmood, S., Corradi, V., Choudhury, H.G., Hussain, R., Becker, P., Axford, D., Zirah, S., Rebuffat, S., Tieleman, D.P., Robinson, C.V., et al. (2016). Structural and functional basis for lipid synergy on the activity of the antibacterial peptide ABC transporter McjD. J. Biol. Chem. *291*, 21656–21668.

Michaelevski, I., Eisenstein, M., and Sharon, M. (2010). Gas-phase compaction and unfolding of protein structures. Anal. Chem. *82*, 9484–9491.

Norimatsu, Y., Hasegawa, K., Shimizu, N., and Toyoshima, C. (2017). Protein–phospholipid interplay revealed with crystals of a calcium pump. Nature *545*, 193–198.

Opekarova, M., and Tanner, W. (2003). Specific lipid requirements of membrane proteins—a putative bottleneck in heterologous expression. Biochim. Biophys. Acta *1610*, 11–22.

Pantazopoulou, A., Lemuh, N.D., Hatzinikolaou, D.G., Drevet, C., Cecchetto, G., Scazzocchio, C., and Diallinas, G. (2007). Differential physiological and developmental expression of the UapA and AzgA purine transporters in *Aspergillus nidulans*. Fungal Genet. Biol. *44*, 627–640.

Parrinello, M., and Rahman, A. (1981). Polymorphic transitions in single crystals: a new molecular dynamics method. J. Appl. Phys. *52*, 7182–7190.

Patrick, J.W., Boone, C.D., Liu, W., Conover, G.M., Liu, Y., Cong, X., and Laganowsky, A. (2018). Allostery revealed within lipid binding events to membrane proteins. Proc. Natl. Acad. Sci. USA *115*, 2976–2981.

Peng, T., Yuan, X., and Hang, H.C. (2014). Turning the spotlight on protein-lipid interactions in cells. Curr. Opin. Chem. Biol. *21*, 144–153.

Politis, A., Park, A.Y., Hyung, S.-J., Barsky, D., Ruotolo, B.T., and Robinson, C.V. (2010). Integrating ion mobility mass spectrometry with molecular modelling to determine the architecture of multiprotein complexes. PLoS One *5*, e12080.

Reading, E., Walton, Troy A., Liko, I., Marty, Michael T., Laganowsky, A., Rees, Douglas C., and Robinson, Carol V. (2015). The effect of detergent, temperature, and lipid on the oligomeric state of MscL constructs: insights from mass spectrometry. Chem. Biol. *22*, 593–603.

Ruotolo, B.T., Benesch, J.L.P., Sandercock, A.M., Hyung, S.-J., and Robinson, C.V. (2008). Ion mobility–mass spectrometry analysis of large protein complexes. Nat. Protoc. *3*, 1139–1152.

Sali, A., and Blundell, T.L. (1993). Comparative protein modelling by satisfaction of spatial restraints. J. Mol. Biol. *234*, 779–815.

Schneiter, R., Brugger, B., Sandhoff, R., Zellnig, G., Leber, A., Lampl, M., Athenstaedt, K., Hrastnik, C., Eder, S., Daum, G., et al. (1999). Electrospray ionization tandem mass spectrometry (ESI-MS/MS) analysis of the lipid molecular species composition of yeast subcellular membranes reveals acyl chain-based sorting/remodeling of distinct molecular species en route to the plasma membrane. J. Cell Biol. *146*, 741–754.

Shvartsburg, A.A., and Jarrold, M.F. (1996). An exact hard-spheres scattering model for the mobilities of polyatomic ions. Chem. Phys. Lett. *261*, 86–91.

Skruzny, M., Desfosses, A., Prinz, S., Dodonova, S.O., Gieras, A., Uetrecht, C., Jakobi, A.J., Abella, M., Hagen, W.J.H., Schulz, J., et al. (2015). An organized Co-assembly of clathrin adaptors is essential for endocytosis. Dev. Cell *33*, 150–162.

van den Brink-van der Laan, E., Chupin, V., Killian, J.A., and de Kruijff, B. (2004). Stability of KcsA tetramer depends on membrane lateral pressure. Biochemistry *43*, 4240–4250.

Wang, S.C., Politis, A., Di Bartolo, N., Bavro, V.N., Tucker, S.J., Booth, P.J., Barrera, N.P., and Robinson, C.V. (2010). Ion mobility mass spectrometry of two tetrameric membrane protein complexes reveals compact structures and differences in stability and packing. J. Am. Chem. Soc. *132*, 15468–15470.

Wöhlert, D., Grötzinger, M.J., Kühlbrandt, W., and Yildiz, Ö. (2015). Mechanism of Na^+-dependent citrate transport from the structure of an asymmetrical CitS dimer. eLife *4*, e09375.

Yefremova, Y., Melder, F.T.I., Danquah, B.D., Opuni, K.F.M., Koy, C., Ehrens, A., Frommholz, D., Illges, H., Koelbel, K., Sobott, F., et al. (2017). Apparent activation energies of protein-protein complex dissociation in the gas-phase determined by electrospray mass spectrometry. Anal. Bioanal. Chem. *409*, 6549–6558.

Zhou, M., Morgner, N., Barrera, N.P., Politis, A., Isaacson, S.C., Matak-Vinkovic, D., Murata, T., Bernal, R.A., Stock, D., and Robinson, C.V. (2011). Mass spectrometry of intact V-type ATPases reveals bound lipids and the effects of nucleotide binding. Science *334*, 380–385.

STAR★METHODS

KEY RESOURCES TABLE

REAGENT or RESOURCE	SOURCE	IDENTIFIER
Chemicals, Peptides, and Recombinant Proteins		
1,2-dioleoyl-*sn*-glycero-3-phosphocholine	Avanti Polar Lipids	Cat #850375
1,2-dioleoyl-*sn*-glycero-3-phosphoethanolamine	Avanti Polar Lipids	Cat #850725
1,2-dioleoyl-*sn*-glycero-3-phospho-(1'-rac-glycerol) (sodium salt)	Avanti Polar Lipids	Cat #840475P
1,2-dioleoyl-*sn*-glycero-3-phospho-(1'-myo-inositol) (ammonium salt)	Avanti Polar Lipids	Cat #850149
Radiolabelled [^{3}H]-Xanthine	Moravek Biochemicals, CA, USA	N/A
Ethylenediamine diacetate	Sigma-Aldrich	CAS: 38734-69-9
Deposited Data		
UapA Structure	(Alguel et al., 2016)	PDB: 5I6C
UapA Sequence	Uniprot	Q07307 (UAPA_EMENI)
LC-MS lipid identification, see Table S1	This paper	N/A
Experimental Models: Organisms/Strains		
Saccharomyces cerevisiae FGY217	(Kota et al., 2007)	N/A
Aspergillus nidulans WT *pabaA1*	(Krypotou and Diallinas, 2014)	N/A
Aspergillus nidulans uapA⊿ uapC⊿ azgA⊿ pabaA1 argB2	(Pantazopoulou et al., 2007).	N/A
Software and Algorithms		
PyMOL	PyMOL Molecular Graphics System, Schrödinger, LLC	https://pymol.org/2/
PULSAR	(Allison et al., 2015)	http://pulsar.chem.ox.ac.uk
MOBCAL	(Shvartsburg and Jarrold, 1996)	http://www.indiana.edu/~nano/software/)
MassLynx v4	Waters Corporation	http://www.waters.com/waters/en_GB/MassLynx-Mass-Spectrometry-Software-/nav.htm?cid=513164&locale=en_GB
UniDec	(Marty et al., 2015)	http://unidec.chem.ox.ac.uk
GROMACS 4.6	(David Van Der et al., 2005)	http://www.gromacs.org
MODELLER	(Fiser and Šali, 2003; Sali and Blundell, 1993)	https://salilab.org/modeller/
Martini 2.2 force-field	(de Jong et al., 2013; Marrink et al., 2007)	http://cgmartini.nl/index.php/224-m22

CONTACT FOR REAGENT AND RESOURCE SHARING

Further information and requests for resources and reagents should be directed to and will be fulfilled by the Lead Contact, Argyris Politis (argyris.politis@kcl.ac.uk).

METHOD DETAILS

Expression of the UapA Constructs and Membrane Preparation

Wild-type (WT) UapA, a thermostable construct of UapA (UapAG411V$_{\Delta 1\text{-}11}$), and a construct of UapA with disrupted lipid binding (R287A/R478A/R479A with G411V$_{\Delta 1\text{-}11}$) (RRR/A + G411V$_{\Delta 1\text{-}11}$) were recombinantly expressed as described previously (Alguel et al., 2016). In brief, *S. cerevisiae* FGY217 cells (6 or 12L) containing one of the UapA constructs were grown at 30°C and shaking at 300 rpm to an OD$_{600}$ of 0.6. Expression was induced by the addition of galactose to a final concentration of 2%. After 22 hr incubation at 30°C with shaking the cells were harvested by centrifugation and resuspended in 10 mL cell resuspension buffer (50 mM

Tris, pH 7.6, 1 mM EDTA, 0.6 M sorbitol) per L culture. The cell suspension was flash frozen and stored at -80°C. Cells were lysed with a Constant Systems cell disruptor at 4°C. Unbroken cells and aggregates were removed by centrifugation at 4000 g for 10 minutes and membranes were isolated by ultracentrifugation at 100,000 g for 2 hrs. Membranes were resuspended in membrane resuspension buffer (20 mM Tris, pH 7.5, 0.3 M sucrose, 0.1 mM $CaCl_2$), flash frozen, and stored at -80°C.

Purification of UapA

Wild-type (WT) UapA, UapA RRR/A + G411V$_{\Delta 1\text{-}11}$, and UapAG411V$_{\Delta 1\text{-}11}$ were purified as described previously (Alguel et al., 2016). In brief, membranes were solubilised for 1 hr at 4°C in membrane solubilisation buffer (1x PBS, pH 7.5, 10% glycerol (v/v), 1 mM xanthine, 100 mM NaCl, 1% n-dodecyl-β-D-maltoside (DDM$_{LA}$) (v/v)) supplemented with 1 complete protease inhibitor tablet (Roche). Non-solubilised material was removed by centrifugation at 100,000 g for 45 min. The supernatant was incubated for 2 hr at 4°C with Ni^{2+}-NTA superflow resin (Qiagen) equilibrated with Affinity buffer (1 x PBS, pH 7.5, 10% glycerol (v/v), 1 mM xanthine, 100 mM NaCl, 0.03% DDM$_{LA}$ (v/v)) supplemented with 10 mM imidazole. The resin was packed onto a chromatography column and washed with 10 column volumes (CVs) Affinity buffer and then 30 CVs of Affinity buffer supplemented with 30 mM imidazole. UapA was eluted from the column with 5 CV Elution buffer (1x PBS, pH 7.5, 10% glycerol (v/v), 1 mM xanthine, 150 mM NaCl, 0.03% DDM$_{LA}$ (v/v), 250 mM imidazole). Tobacco Etch Virus (TEV) protease was added to the protein at a protease:UapA ratio of 1:1, and the sample was dialysed overnight into Dialysis buffer (20 mM Tris, pH 7.5, 5% glycerol (v/v), 0.6 mM xanthine, 150 mM NaCl, 0.03% DDM$_{LA}$ (v/v)). The dialysed protein sample was loaded onto a 5 mL His-trap column (G.E. Biosciences) and UapA was recovered mainly in the initial flow through with any residual UapA eluted by washing the His-trap column with 5 CVs Reverse Affinity buffer (20 mM Tris, pH 7.5, 0.6 mM xanthine, 150 mM NaCl, 0.03% DDM$_{LA}$ (v/v), 10 mM imidazole). The His-tagged GFP and His-tagged TEV remained bound to the column under these conditions. The UapA sample was then loaded onto a Superdex 200 10/300 gel filtration column pre-equilibrated with SEC buffer (20mM Tris, pH 7.5, 0.6mM xanthine, 150mM NaCl, 2x critical micellar concentration (CMC) of the detergent of interest). Fractions were analysed by SDS-PAGE. Fractions containing UapA were concentrated to ~ 9mg/mL. Protein solution was flash frozen and stored in small aliquots of 10μL at -80°C.

Native MS

Capillaries for nESI were prepared using a Model P-97 (Sutter Instruments) capillary puller. Capillaries were gold coated using a Q150R S sputter coater (Quorum). Using Micro Bio-Spin 6 columns (Bio-Rad) purified UapA was buffer-exchanged into MS-compatible buffer (250 mM EDDA (pH 6.3), 0.014% DDM$_{LA}$ (v/v), 10 mM L-serine) to a final protein concentration of 15 μM. The MS buffer was supplemented with 10 mM L-serine to improve spectral resolution by minimising salt adducts (Clarke and Campopiano, 2015). Delipidated samples were prepared by incubating purified UapA with 1% DDM$_{LA}$ (v/v) overnight before removing displaced lipids and buffer-exchanging UapA into the MS-compatible buffer using Micro Bio-Spin 6 columns (Bio-Rad). The individual proteins samples were loaded into gold-coated nanoflow capillaries (Hernandez and Robinson, 2007) and introduced into a Synapt G2-Si (Waters) mass spectrometer by nESI. The conditions for maximum peak resolution for UapA were: capillary voltage +1.2-1.6 kV, sampling cone voltage 5 V, trap collision energy (CE) 125-200 V, transfer CE 50-240 V, backing pressure 3.88 mbar, trap and transfer pressure (argon) $1.72e^{-2}$ mbar, ion mobility cell pressure (nitrogen) 2.58 mbar. A trap CE of 200 V and a transfer CE of 240 V was used when determining the relative abundances of each oligomeric species. To identify lipids in the protein sample, a negative capillary voltage of -1.1 kV was used. Mass measurements were calibrated using caesium iodide (100 mg/mL). Spectra were recorded and smoothed using Masslynx 4.1 (Waters) software.

Lipid Titrations

Phosphatidylcholine (PC) (34:1), phosphatidylethanolamine (PE) (34:1), phosphatidylinositol (PI) (34:1), and phosphatidylglycerol (PG) (34:1) were supplied by Avanti Polar Lipids. Liposomes were prepared by dissolving lipid to 25 mg/mL in chloroform before evaporating the solvent under a N_2 stream. The lipid film was frozen using liquid N_2 and placed under vacuum in a freeze-dryer (Labconco Freezone) overnight. Lipids were solubilised to a concentration of 3 mM in 250 mM EDDA, 0.014% DDM$_{LA}$ (v/v), 10 mM L-serine. Lipids were homogenised by 30 minutes sonication followed by three rounds of freeze-thawing. Lipids were added to delipidated UapA after buffer exchange into the MS-compatible buffer to a final concentration of 1.5mM lipid and 15μM UapA. Lipids were incubated with UapA for at least 1.5 hrs before analysis by native MS under the following conditions: capillary voltage +1.2-1.6 kV, sampling cone voltage 5 V, trap CE 200 V, transfer CE 240 V, backing pressure 3.88 mbar, trap and transfer pressure (argon) $1.72e^{-2}$ mbar, ion mobility cell pressure (nitrogen) 2.58 mbar.

The relative abundances of each oligomeric state and lipid bound state of UapA was calculated using UniDec, a spectrum deconvolution software package (Marty et al., 2015), after correcting peak intensity by accounting for detector efficiency (Fraser, 2002). Spectra were smoothed using MassLynx 4.1 (Waters) software prior to deconvolution. The mass range for peak detection was 60000-126000 Da. The following parameters were adjusted between samples to minimise the assignment of background noise as UapA species: charge (minimum of 8, maximum of 29-32), mass (monomer: 61025 ± 25, dimer 122025 ± 25, dimer + lipid 122775 ± 50, dimer + two lipids 123550 ± 50 Da), and intensity threshold (0.00 to 0.45). This method assumes all UapA species have similar ionisation efficiencies.

IM-MS

Conditions in the mass spectrometer for IM-MS of the dimer were: capillary voltage +1.2-1.6 kV, sampling cone voltage 5 V, trap CE 20 V, transfer CE 200 V, capillary backing pressure 0 bar. Drift times were measured at a T-wave height of 40 V and at three T-wave velocities (550, 600, 640 m/s). The following range of calibrants were utilised at 10 μM in 200 mM ammonium acetate: concanavalin A, β-lactoglobulin, pyruvate kinase, glutamate dehydrogenase and alcohol dehydrogenase. PULSAR software (available from http://pulsar.chem.ox.ac.uk/) (Allison et al., 2015) was used to create a calibration curve (Bush et al., 2010) and calculate CCS values for UapA. MOBCAL software (available from http://www.indiana.edu/~nano/software/) was used to calculate the CCS of the UapA crystal structure (PDB: 5I6C) (Shvartsburg and Jarrold, 1996). The CCS of missing residues from the crystal structure was accounted for using the following equation (Hall et al., 2012; Politis et al., 2010):

$$CCS_{Total} = 1.14 * CCS_{PA} * \left(\frac{Mass_{MS}}{Mass_{PDB}}\right)^{\frac{2}{3}}$$

Equation 1: CCS_{PA} refers to the CCS calculated using the proximal approximation via MOBCAL. $Mass_{MS}$ refers to the mass of the protein analysed by MS. $Mass_{PDB}$ refers to the mass of protein in the PDB file.

LC-MS

A 10 μL 60 μM UapA sample was subjected to a lipid extraction as described by the Folch method (Folch et al., 1957). The lipid extract was separated by liquid chromatography using an Accela Autosampler (Thermo Scientific) coupled to a LTQ Orbitrap Elite (Thermo Scientific) mass spectrometer. 5 μL lipid extract was separated at a flow rate of 0.5 mL/min on an Acuity C18 BEH column (Waters, 50x2.1mm, 1.7μm) at 55°C. Mobile phase A = acetonitrile:water (60:40) with 10mM ammonium acetate. Mobile phase B = isopropanol:acetonitrile (90:10) with 10mM ammonium acetate. Lipids were initially separated with 60:40 mobile phase A:B. This mobile phase gradient was linearly changed to 1:99 A:B over 8 minutes and kept constant for 30 seconds. 60:40 mobile phase A:B was then regained in 10 seconds. These conditions were maintained for a further 2.5 minutes. Lipids were analysed by MS in the negative ion mode. Tandem MS was performed to fragment intact lipids in order to identify the fatty acyl chains using their diagnostic ions and the LIPID MAPS database (Fahy et al., 2007). Cardiolipin also co-purified with $UapAG411V_{\Delta 1\text{-}11}$ but is likely to be a contaminant from the yeast mitochondria (Schneiter et al., 1999).

Coarse-grained Molecular Dynamics (CG-MD) Simulations

The CG-MD simulations were performed using the Martini 2.2 force-field (de Jong et al., 2013; Marrink et al., 2007) and GROMACS 4.6 (David Van Der et al., 2005). The crystal structure of UapA dimer (PDB: 5I6C) (Alguel et al., 2016) (residues 66 to 545) was used for the CG-MD simulations. Missing unstructured regions from the crystal structure were added using Modeller (Fiser and Šali, 2003; Sali and Blundell, 1993) prior to the simulations and the V411 mutation in the crystal was mutated to glycine to generate a WT model. We also note that prior to the conversion to the coarse-grained representation, the substrate, xanthine, present in the crystal structure was removed.

A POPC bilayer was self-assembled around the UapA dimer and the snapshot at the end of this simulation was taken. Six different systems were generated with the protein inserted in complex symmetric bilayers that contained the following lipid concentrations: 1) 100% POPC, 2) 100% POPE, 3) 60% POPC-40% POPE, 4) 40% POPC-25% POPE-35% PI, 5) 65% POPC-25% POPE-10% PI, and 6) 70% POPC-25% POPE-5% PI. The exchange of lipids was done as previously described (Koldsø et al., 2014). For the simulations, we have used CG models of POPC and POPE lipids that had 4 CG particles in one of the lipids tails and 5 CG particles in the other tail (with one particle representing the double bond in the chain with 5 particles). Those are the CG-equivalent of the 1-palmitoyl 2-oleoyl-phosphatidylcholine (POPC) and 1-palmitoyl 2-oleoyl-phosphatidylethanolamine (POPE). The PI lipids had a tail with 4 CG particles and a tail with 5 CG particles but with no double bond. CG water particles were added to solvate the simulation systems and ~150 nM of NaCl was added to neutralize the systems. The systems were equilibrated for 10 ns with the protein backbone particles restrained. We ran simulations with both the dimer interface restrained with an ENM network and without restraining the dimer interface. For simulation systems 1 to 5 we have performed 5 repeat simulations in which an elastic network using a cut-off distance of 7 Å was used for the complete dimer and 5 repeat simulations in which the elastic network was applied to each UapA monomer. For simulation system 6 we have performed simulations in which the elastic network was applied to each UapA monomer.

The integration step was 20 fs. All simulations were run for 5 μs each. The temperature was set to 323 K. The V-rescale thermostat (Bussi et al., 2007) (coupling constant of 1.0) was used for temperature control. A Parrinello-Rahman barostat (Parrinello and Rahman, 1981) (a coupling constant of 1.0 and a reference pressure of 1 bar) was used for pressure control. Lennard-Jones and Coulombic interactions were shifted to zero between 9 and 12 Å, and between 0 and 12 Å, respectively.

A. nidulans Growth Conditions, UapA Localisation, Bifluorescence Complementation Assays and Xanthine Transport Assays

An *A. nidulans* mutant strain (*uapAΔ uapCΔ azgAΔ pabaA1 argB2*) lacking the genes encoding all major endogenous purine uptake systems was used (Pantazopoulou et al., 2007). This strain will only grow using xanthine or uric acid as the sole nitrogen source when a functional UapA construct is introduced. GFP-tagged UapA constructs ($UapA^{+}$ (WT), R287A, R478A, R479A, R478A/R479A, R287A/R478A/R479A) were generated and transformed as previously described (Koukaki et al., 2003) into *A. nidulans*. The GFP

tag has been shown not to affect UapA localisation, function, or transport kinetics in *A. nidulans* (Pantazopoulou et al., 2007). Successfully transformed strains of *A. nidulans* were selected via arginine auxotrophy complementation (Pantazopoulou et al., 2007).

As previously described (Karachaliou et al., 2013), UapA subcellular localisation was measured by visualising GFP-tagged UapA using epifluorescence inverted microscopy (Zeiss Observer Z1/Axiocam HR R3 camera/Zen lite 2012 software). Radiolabelled [^{3}H]-xanthine (22.8 Ci mmol^{-1}, Moravek Biochemicals, CA, USA) uptake was measured using *A. nidulans* germinating conidiospores at 37°C or 25°C, pH 6.8, as previously described (Krypotou and Diallinas, 2014). To measure growth of *A. nidulans* in different nitrogen sources, transformed strains were grown on minimal media supplemented with nitrogen sources (10 mM ammonium tartrate or 0.5 mM uric acid or 0.5 mM xanthine) at 37°C or 25°C, pH 6.8. Dimerisation of mutant UapA was measured via a bimolecular fluorescence complementation assay, as described previously (Martzoukou et al., 2015). In brief, the N-terminal 154 amino acids of YFP or the C-terminal 86 amino acids of YFP were cloned into the pAN510exp or pAN520exp vector at the XbaI site. uapA with the necessary mutations was then cloned into the vector and transformed into *A. nidulans*. YFP fluorescence was measured using epifluorescence inverted microscopy (Zeiss Observer Z1/Axiocam HR R3 camera/Zen Lite 2012 software). Relative quantification was carried out using the ICY colocaliszation studio plugin (pixel-based method) (http://icy.bioimageanalysis.org/) and statistical analysis (Tukey's Multiple Comparison Test, One-Way ANOVA for n=5 hyphae) of plasma membrane fluorescence intensity of mutants compared to WT UapA+-YFPC/UapA+-YFPN was performed as previously described (Martzoukou et al., 2017).

QUANTIFICATION AND STATISTICAL ANALYSIS

Bar charts throughout show mean ± standard deviation (n=3, where n represents the number of repeats). For the bimolecular fluorescence complementation analysis using a split YFP system (Figure 5B), statistical analysis (Tukey's Multiple Comparison Test, One-Way ANOVA for n=5 hyphae) of plasma membrane fluorescence intensity of mutants compared to WT UapA+-YFPC/UapA+-YFPN was performed as previously described (Martzoukou et al., 2017). No additional statistical tests were undertaken.

Cell Chemical Biology, Volume *25*

Supplemental Information

Structural Lipids Enable the Formation of Functional Oligomers of the Eukaryotic Purine Symporter UapA

Euan Pyle, Antreas C. Kalli, Sotiris Amillis, Zoe Hall, Andy M. Lau, Aylin C. Hanyaloglu, George Diallinas, Bernadette Byrne, and Argyris Politis

Supplementary Figure Titles and Legends

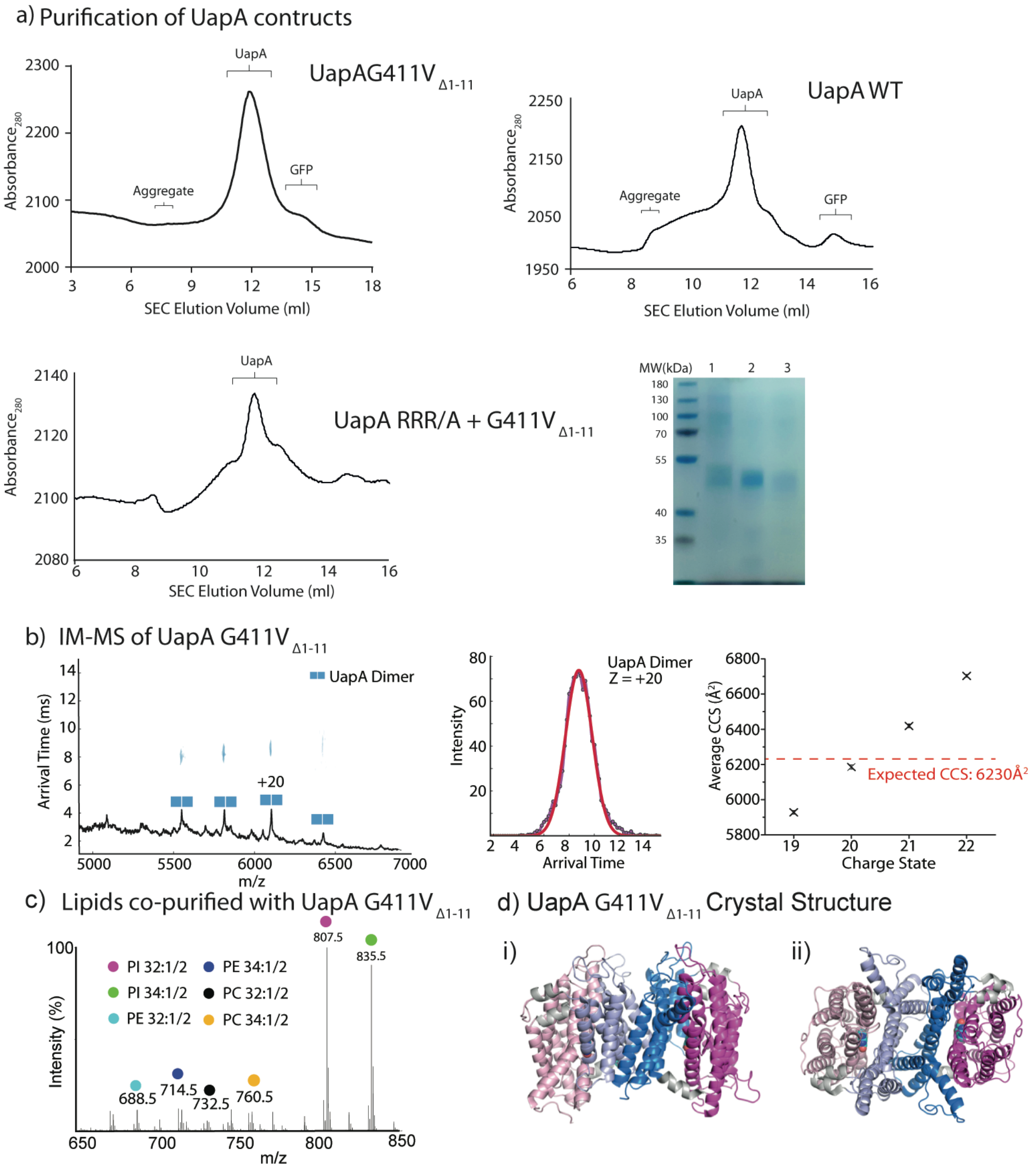

Figure S1, related to Figure 1 a) SEC elution profile of UapAG411V$_{\Delta 1\text{-}11}$, UapA RRR/A + G411V$_{\Delta 1\text{-}11}$, and WT UapA (λ=280 nm) with an SDS-PAGE gel showing the purity of UapA after SEC (lane 1: WT UapA, lane 2: UapAG411V$_{\Delta 1\text{-}11}$, lane 3: UapA RRR/A + G411V$_{\Delta 1\text{-}11}$) (lanes without protein loaded into their respective wells have been digitally cropped) (b) Ion-Mobility MS analysis of the UapAG411V$_{\Delta 1\text{-}11}$ dimer. IM-MS measurements were taken at a trap CE of 20 V and a transfer CE of 200 V. The resulting spectrum (left) is displayed with the arrival time distribution of z = +20 charge state shown (centre). The average CCS of each charge state (n= 3) (± s.d.) was plotted against the expected CCS of UapAG411V$_{\Delta 1\text{-}11}$ calculated by analysing the UapAG411V$_{\Delta 1\text{-}11}$ crystal structure (PDB: 5I6C) (Alguel et al. 2016) using MOBCAL software (Shvartsburg and Jarrold 1996). (c) Negative ion mass spectrum of

UapAG411V$_{\Delta 1\text{-}11}$ in the low m/z range identifying the lipids co-purified with UapAG411V$_{\Delta 1\text{-}11}$. Due to overlapping peaks of mono-unsaturated and poly-unsaturated fatty acid chains, both species are identified by 1/2 notation. d) Structure of UapA (PDB: 5I6C) highlighting the domain organisation of the protein. The UapA dimer is shown (i) looking through the membrane and (ii) from the extracellular side of the membrane. The core domains of the individual monomers are coloured in light pink and magenta and the gate domains are coloured in light blue and dark blue. The amphipathic helices connecting the core and gate domains are coloured in gray and xanthine is shown in a space filling representation with cyan coloured carbon atoms.

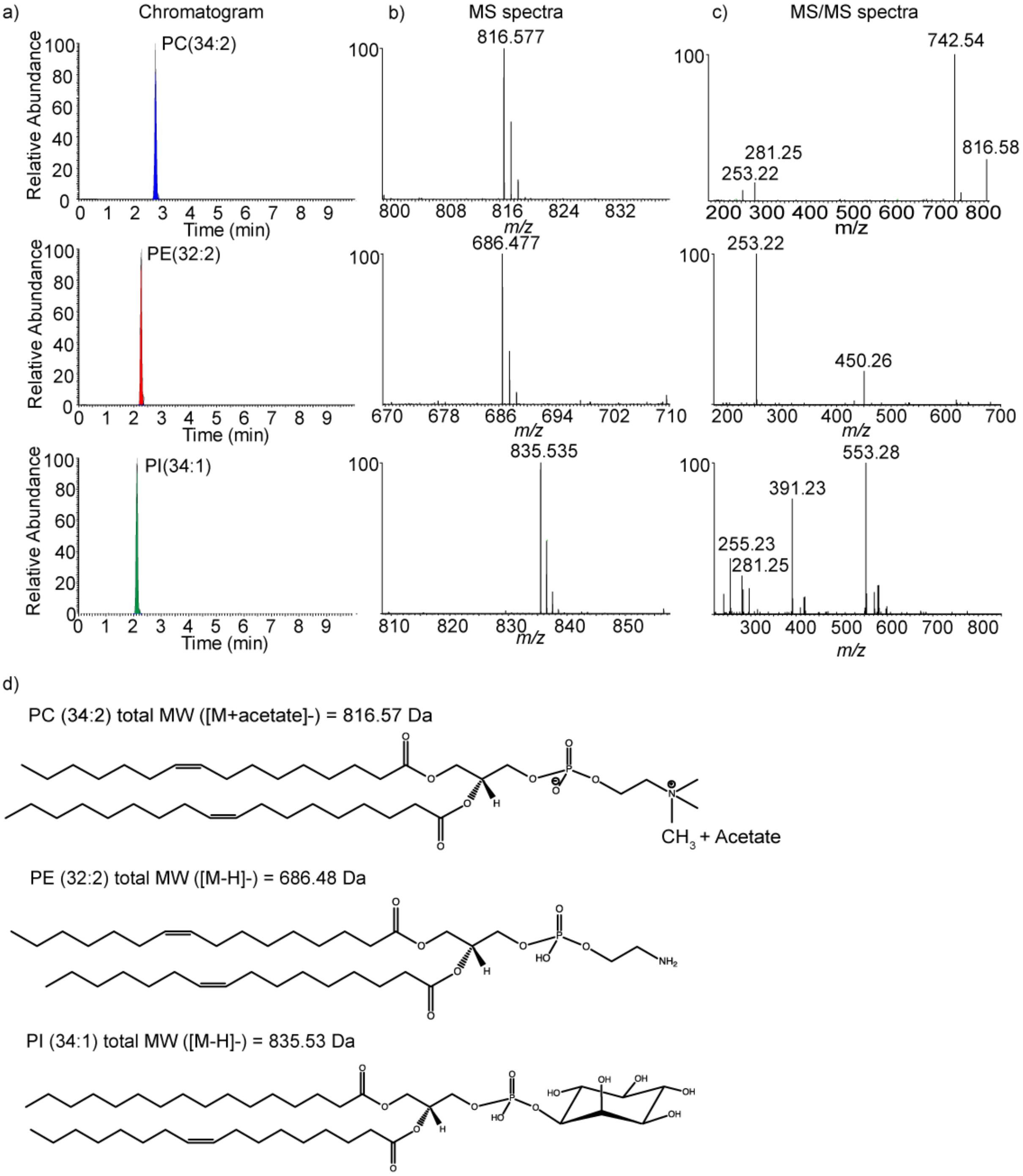

Figure S2, related to Figure 3 Results from LC-MS(/MS) analysis of UapAG411V$_{\Delta 1\text{-}11}$. LC traces (a) show the retention times of each major lipid species identified. The corresponding MS spectra (b) from the precursor ions of each lipid species are also displayed. LC-MS/MS spectra (c) show the fragmentation patterns of the lipid precursor ions. d) Molecular structure for each major lipid species identified by LC-MS(/MS). Also see **Table S1**.

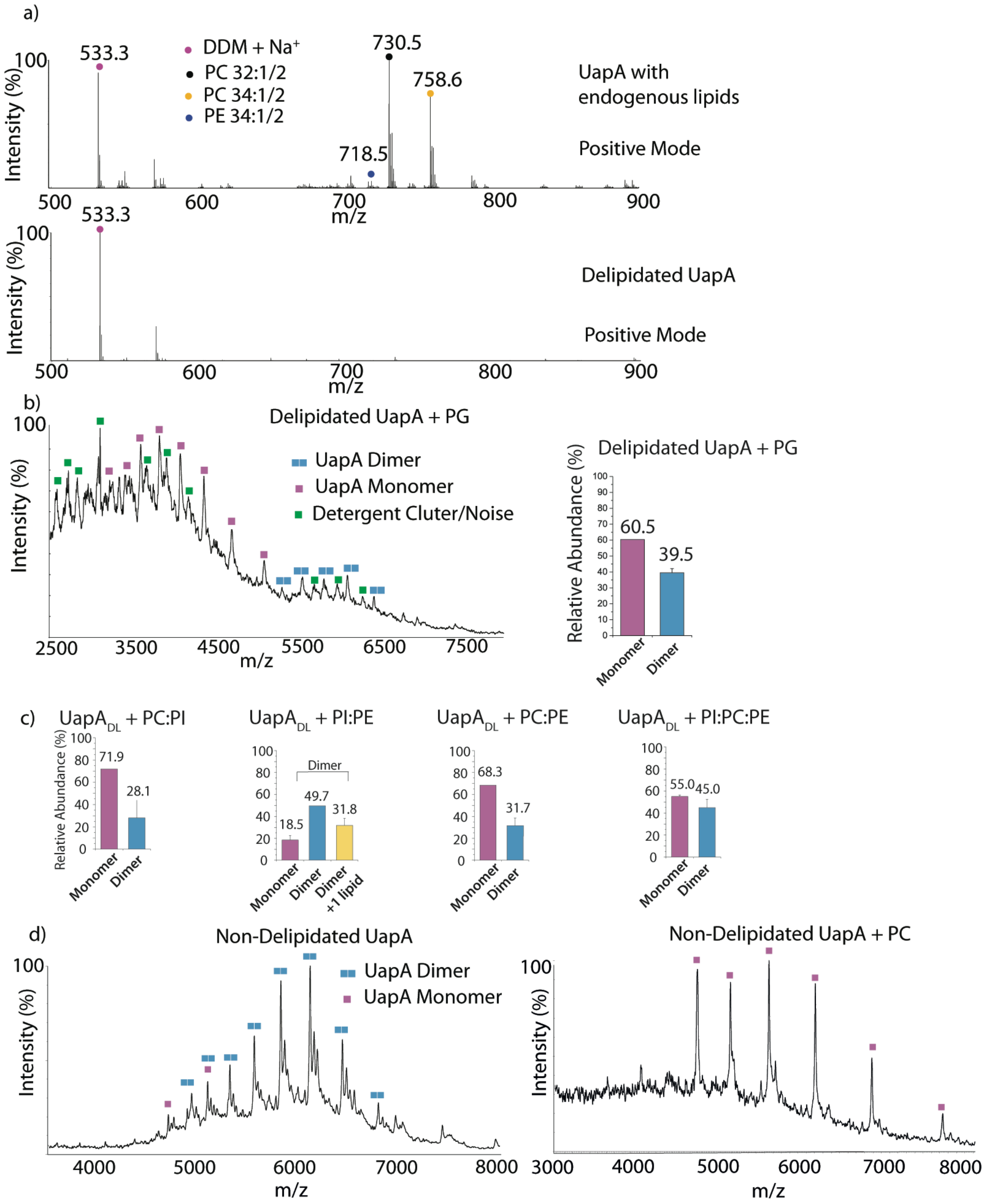

Figure S3, related to Figure 2 a) Spectra showing the presence of lipids in the low m/z range of a non-delipidated UapAG411V$_{\Delta 1\text{-}11}$ sample (top panel) in native MS and the loss of lipids in the same m/z range for a UapAG411V$_{\Delta 1\text{-}11}$ sample after delipidation (bottom panel). These spectra were recorded at a trap CE of 200 V and a transfer CE of 240 V. (b) Spectrum showing the effect of adding PG to delipidated UapAG411V$_{\Delta 1\text{-}11}$ at a ratio of 100:1 PG:UapA. The

relative abundance of each oligomeric state (right panel) of UapAG411V$_{\Delta 1\text{-}11}$ has been calculated using UniDec software. Data shown is the average ± s.d. Data is representative of three independent experiments carried out under identical conditions. This spectrum was recorded at a trap CE of 200 V and a transfer CE of 240 V. (c) Bar charts displaying the relative abundances of UapA species after the addition of equimolar mixtures of lipids to delipidated UapA. The relative abundances of each oligomeric species were quantified using UniDec software. Data shown is the average ± s.d. Data is representative of three independent experiments carried out under identical conditions. (d) Spectrum showing the effect of adding PC to non-delipidated UapAG411V$_{\Delta 1\text{-}11}$ at a ratio of 100:1 PC:UapA. These spectra were recorded at a trap CE of 200 V and a transfer CE of 240 V.

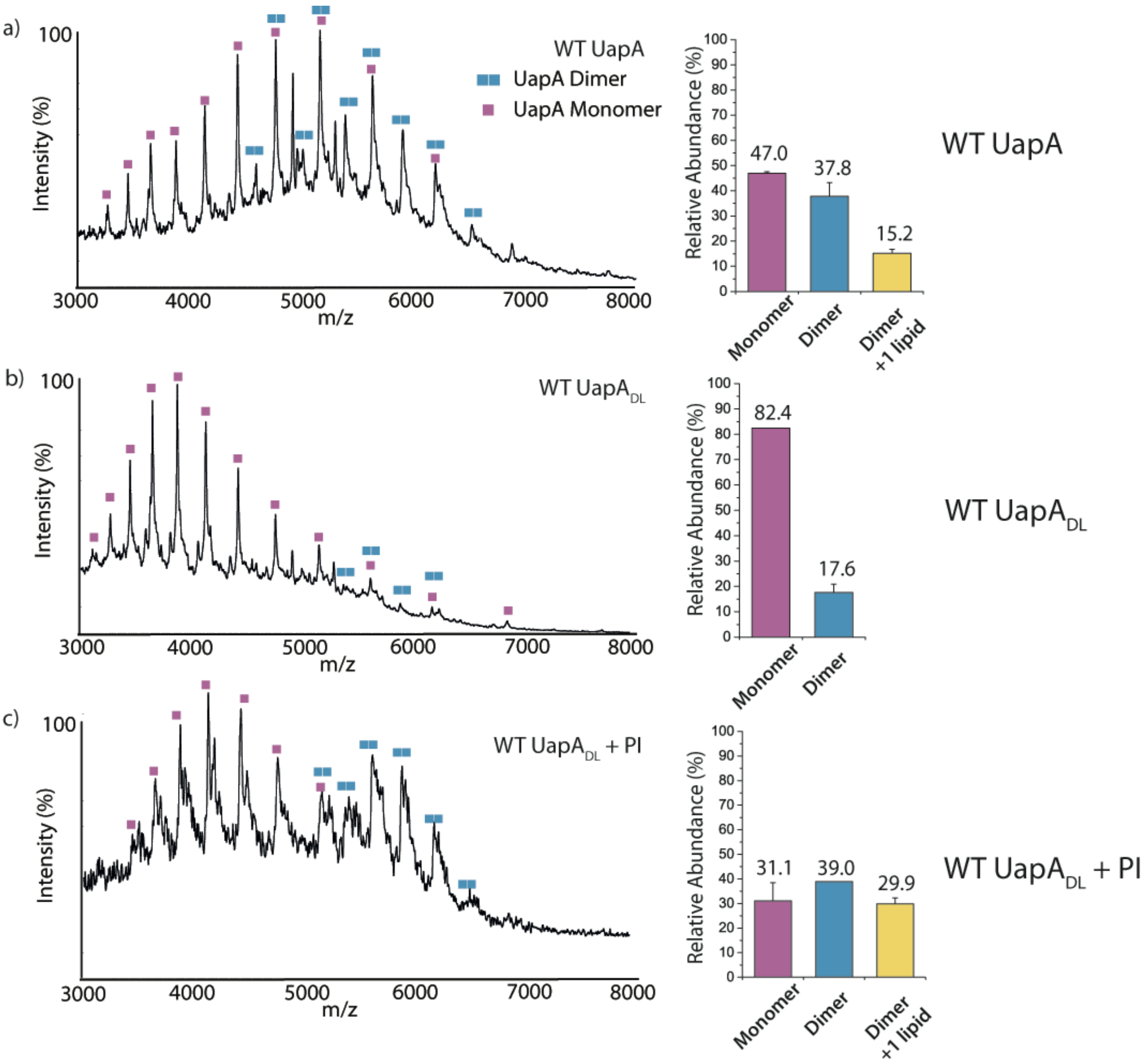

Figure S4, related to Figure 2 (a) Analysis of WT UapA, (b) delipidated WT UapA and (c) delipidated WT UapA following addition of PI. The left-hand panels show the spectra for the individual samples and the right hand panels show the relative abundance of the different oligomeric states obtained in each case. The spectra were recorded at a trap CE of 200 V and a transfer CE of 240 V. Quantification of the relative abundance of each oligomeric species was carried out using UniDec software. Data shown is the average ± s.d. Data is representative of three independent experiments carried out under identical conditions.

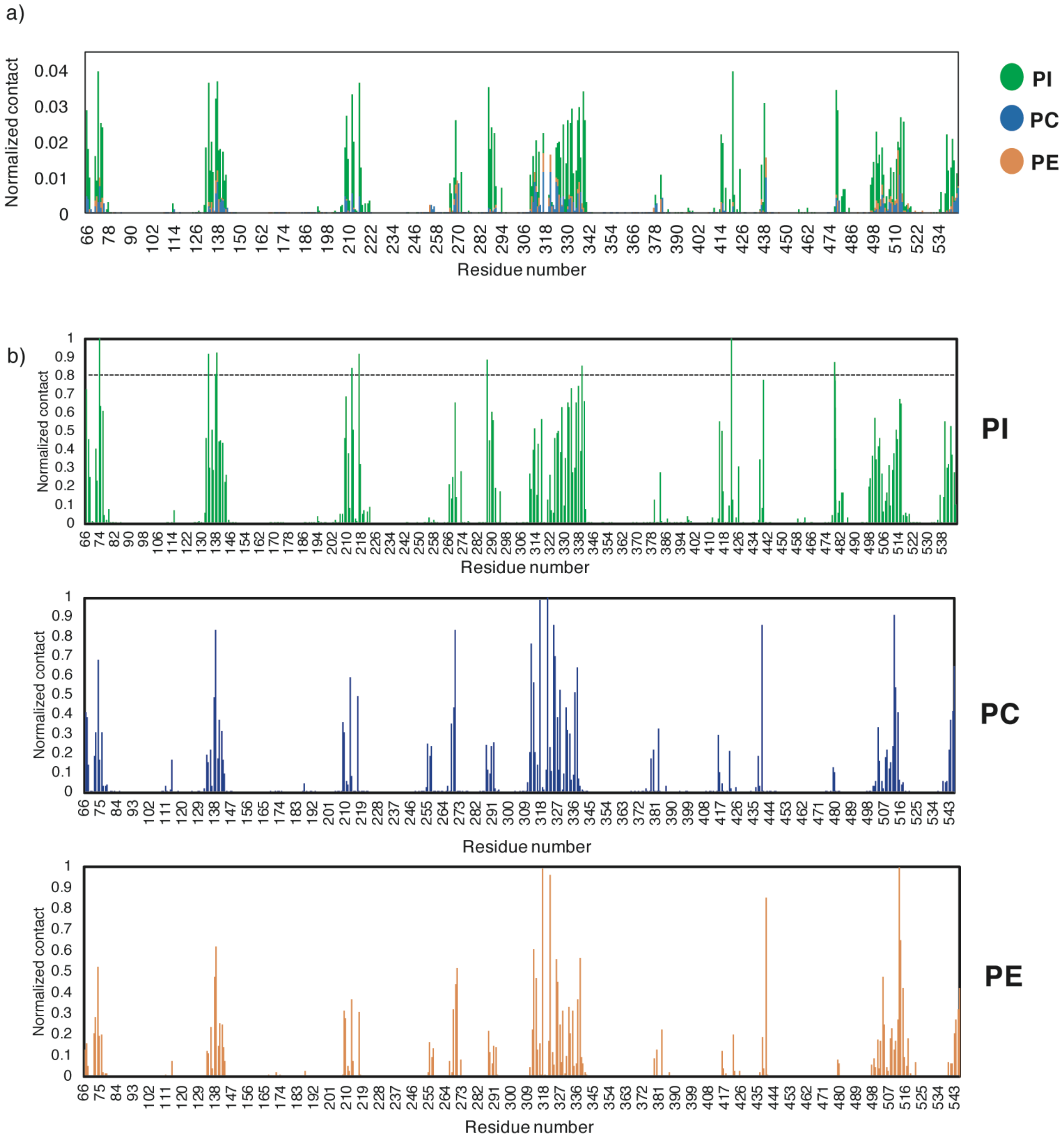

Figure S5, related to Figure 3: (a) Normalised average number of contacts (using a cut-off distance of 5.5 Å) between each UapA residue and the head groups of PI, PE, and PC lipids in the lipid bilayer in our MD simulations with a 40% POPC-25% POPE-35% PI bilayer (no restraints in the UapA interface). Each peak represents the probability of a residue to interact with each lipid type over the 5 μs of the simulations. For the normalization, the number of contacts of a residue with a lipid type was divided by the number of lipids, the number of frames and the number of lipids in the simulation. (b) Normalised average number of contacts between each UapA residue and the head groups of PI, PE, and PC lipids in the lipid bilayer of

our MD simulations. For the normalization, the number of contacts of a residue with the lipids was divided by the largest number of contacts, the number of frames and the number of lipids in the simulation.

Lipid	Acyl chains	Species	Lipid Mass (Neutral)	**Mean Lipid Abundance (mol) (%)**
PC(32:2)	16:1/16:1	$[M+acetate]^-$	729.5309	**13.6 ± 2.8**
PC(32:1)	16:0/16:1	$[M+acetate]^-$	731.5465	**6.6 ± 3.1**
PC(34:1)	16:0/18:1	$[M+acetate]^-$	759.5778	**4.3 ± 1.7**
PC(34:2)	16:1/18:1	$[M+acetate]^-$	757.5622	**10.9 ± 1.8**
PE(32:1)	16:0/16:1	$[M-H]^-$	689.4996	**6.0 ± 2.7**
PE(32:2)	16:1/16:1	$[M-H]^-$	687.4839	**4.1 ± 0.4**
PE(34:1)	16:0/18:1	$[M-H]^-$	717.7309	**6.6 ± 1.8**
PE(34:2)	16:1/18:1	$[M-H]^-$	715.5152	**5.5 ± 2.0**
PE(36:2)	18:1/18:1	$[M-H]^-$	743.5465	**3.0 ± 1.3**
PI(32:1)	16:0/16:1	$[M-H]^-$	808.5102	**10.5 ± 1.6**
PI(32:2)	16:1/16:1	$[M-H]^-$	806.4945	**3.5 ± 1.4**
PI(34:1)	16:0/18:1	$[M-H]^-$	836.5415	**13.9 ± 5.2**
PI(34:1)	16:1/18:1	$[M-H]^-$	834.5258	**4.6 ± 2.3**
PI(36:1)	18:0/18:1	$[M-H]^-$	864.5728	**1.9 ± 1.5**
CL(68:4)	16:1/16:1/18:1/18:1	$[M-H]^-$	1400.9722	**1.6 ± 0.9**
CL(68:4)	16:1/16:1/18:1/18:1	$[M-2H]^{2-}$	1400.9722	**2.0 ± 1.1**
CL(68:4)	16:1/16:1/18:1/18:1	$[M-2H+Na]^-$	1400.9722	**0.7 ± 0.7**
CL(70:4)	16:1/18:1/18:1/18:1	$[M-H]^-$	1429.0035	**0.4 ± 0.4**
CL(72:4)	18:1/18:1/18:1/18:1	$[M-H]^-$	1457.0348	**0.4 ± 0.4**

Green	Confirmed by MS/MS
Orange	Some diagnostic ions detected by MS/MS
Red	No diagnostic ions detected by MS/MS

Table S1, related to Figure 1: Table containing all lipids identified in a UapAG411V$_{\Delta 1\text{-}11}$ sample by LC-MS. Lipids confirmed by LC-MS/MS fragmentation are highlighted in green, lipids with incomplete fragmentation patterns in LC-MS/MS are highlighted in orange, and lipids with no matching fragmentation patterns in LC-MS/MS are highlighted in red. Lipid abundances displayed are the average $\pm$ s.d (n=3)
